# Deep learning-based object detection algorithms in medical imaging: Systematic review

**DOI:** 10.1016/j.heliyon.2024.e41137

**Published:** 2024-12-11

**Authors:** Carina Albuquerque, Roberto Henriques, Mauro Castelli

**Affiliations:** NOVA Information Management School, Lisboa, Portugal

**Keywords:** Deep learning, Object detection, Medical imaging, Bibliometric analysis, Qualitative analysis, Quantitative analysis

## Abstract

Over the past decade, Deep Learning (DL) techniques have demonstrated remarkable advancements across various domains, driving their widespread adoption. Particularly in medical image analysis, DL received greater attention for tasks like image segmentation, object detection, and classification. This paper provides an overview of DL-based object recognition in medical images, exploring recent methods and emphasizing different imaging techniques and anatomical applications. Utilizing a meticulous quantitative and qualitative analysis following PRISMA guidelines, we examined publications based on citation rates to explore into the utilization of DL-based object detectors across imaging modalities and anatomical domains. Our findings reveal a consistent rise in the utilization of DL-based object detection models, indicating unexploited potential in medical image analysis. Predominantly within Medicine and Computer Science domains, research in this area is most active in the US, China, and Japan. Notably, DL-based object detection methods have gotten significant interest across diverse medical imaging modalities and anatomical domains. These methods have been applied to a range of techniques including CR scans, pathology images, and endoscopic imaging, showcasing their adaptability. Moreover, diverse anatomical applications, particularly in digital pathology and microscopy, have been explored. The analysis underscores the presence of varied datasets, often with significant discrepancies in size, with a notable percentage being labeled as private or internal, and with prospective studies in this field remaining scarce. Our review of existing trends in DL-based object detection in medical images offers insights for future research directions. The continuous evolution of DL algorithms highlighted in the literature underscores the dynamic nature of this field, emphasizing the need for ongoing research and fitted optimization for specific applications.

## 1 Introduction

### 1.1 Background

Medical imaging is an essential tool, crucial in diagnosis and treatment planning in the medical field. Various approaches, such as Magnetic resonance imaging (MRI), computed tomography (CT), digital mammography, X-ray, ultrasound, positron emission tomography (PET), and digital pathology, are used to generate images that aid in a variety of tasks such as pathology localization, the study of anatomical structure, treatment planning, and even computer-integrated surgery, among others [[1], [2], [3]].

Humans have traditionally performed medical image analysis and interpretation for several years. However, with the rapid advancement of artificial intelligence (AI), the medical field increasingly embraces computer-assisted tools as a resource to solving diagnostic problems with precision and efficiency. This allows for real-time prediction of various diseases and in-depth examination of different treatment options [3,4], avoiding limitations such as inter-observer and intra-observer variability [5] and error resulting from significant variations in pathologies and the possible exhaustion of human experts [6].

The bibliometric analysis allows for uncovering emerging trends and patterns in the scientific knowledge of a specific domain in a quantitative approach [7], by combining disciplines such as information science, mathematics, and statistics [8]. Deep learning application in medical image analysis has been significantly exploited in recent years due to its current relevance. Many bibliometric analysis works have focused on various fields of research including the usage of artificial intelligence in healthcare [9], medical image segmentation [10], the application of deep learning in the medical field [11], medical data mining research [12], and machine learning-based disease diagnosis [13]. To the best of our knowledge, bibliometric and qualitative analysis research has not been published on applying object detection algorithms in the medical field.

### 1.2 Research problem and aim

In this work, we propose to conduct a comprehensive quantitative and qualitative analysis of the literature related to applying deep learning object detectors in medical imaging and to reveal potential trends in this field. Our contributions include.

⁃ A bibliometric analysis of the application of deep learning object detectors was conducted using two of the most widely used databases: Scopus and the Web of Science (WoS).
⁃ The reporting of several relevant quantitative indicators, such as annual publication analysis, research area analysis, journal publication analysis, publications by country and author, and keyword analysis.

By conducting and employing bibliometric analysis, we aim to evaluate the overall status of the application of object detection in the medical field and identify possible divergences among distinct screening methods and anatomical areas. Furthermore, our work also reflects on.

• The publications trends on this topic, and concretely the growth trajectory of object detection applications in medical imaging.
• The most influential authors, i.e., the most prolific and leading contributors concerning object detection in medical field research.
• The international collaborations existing in this field.
• The most highly cited papers and the topics they cover.
• The emerging research themes in this topic, how those themes evolved over time, and the ones that gained the most attention in recent years.
• The academic journals representing the main outlets for the research on object detection in the medical field.
• The algorithms, screening methods, and anatomical areas under study that experienced the most significant growth in terms of publications.
• The data sets used, their availability, the sample size of these data sets, whether external or internal data sets are used in the different studies, and the nature of the study (retrospective or prospective).

This research will allow researchers to gain a deeper understanding of the field's development and status and guide future research by identifying potential research gaps and collaborative efforts within the research community.

The top publications were filtered based on annual citation rates and analyzed in greater depth in the qualitative analysis. Additionally, we aim to assess the frequency at which different algorithms are applied within the identified articles, and to identify the imaging modalities and the anatomical application areas where object detectors are used with more incidence.

By assessing the frequency at which different algorithms are applied, we aim to understand the diversity of approaches used in object detection for medical imaging, giving insights into the popularity and effectiveness of various algorithms. The occurrence analysis of the algorithms allows us to identify which approaches are commonly employed, which may lead to better performance, and which areas might require further research.

By identifying the imaging modalities and anatomical regions, we can draw some conclusions concerning areas where future research is needed and identify the related specific challenges and requirements.

The combination of the quantitative analysis with the qualitative approach provides a holistic view of the state of the art in the field. We consider this knowledge valuable for further research, guiding future studies, and supporting decision-making for the development and implementation of object detection techniques in medical settings.

To the best of our knowledge, there have been no prior studies conducted exclusively on the topic of object detectors in medical imaging. We acknowledge that some studies proposed the application of deep learning techniques in the medical field, but focused mainly on classification problems and segmentation problems.

The structure of this work is as follows: in the first section, a brief introduction, background to the problem, and purpose of this work are provided. The following section outlines the research methodology, data collection procedures, bibliometric tools, and screening methods. The third section presents a quantitative and descriptive view supported by a bibliometric analysis of the filtered information. In the fourth section, a qualitative analysis of the most cited papers is conducted, and an exploratory analysis is performed on the incidence of different object detectors applied and the medical fields where object detection is more prevalent. In the final section, the conclusions of this study and the overall findings are presented.

## 2 Materials and methods

We utilized two well-known academic databases to construct a bibliometric analysis that is complemented by a qualitative analysis. The Web of Science (WoS) Core Collection provides researchers with a substantial amount of pertinent publications dating back to 1900, including six Citation Indexes such as the Science Citation Index Expanded (SCIE), in addition to journal citation reports (JCR) and essential science indicators (ESI). The second database is Scopus, which encompasses peer-reviewed journals in the life, physical, social, and health sciences.

The framework for gathering and filtering data was based on the Preferred Reporting Items for Systematic Reviews and Meta-Analyses (PRISMA) guidelines. The query search, conducted on January 20th, 2023, was centered around three main topics that align with our purpose, excluding the concept of segmentation on keywords or titles, and limiting the results to articles written in English, as depicted in the Supplementary File 1. Those results comprise papers up until the end of 2022.

**Fig. 1:**
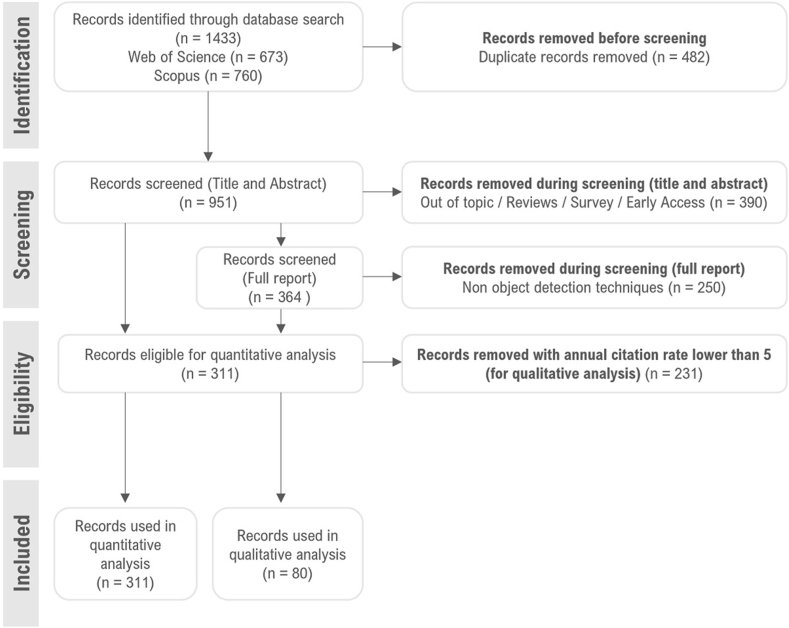
Data collection and filtering process for object detection bibliometric analysis, following PRISMA methodology.

The number of records retrieved from the WoS was 673, and 760 from Scopus. Following the PRISMA methodology, as illustrated in Fig. 1, duplicates were identified, and exclusion criteria were applied.

Removing duplicated articles resulted in a database of 951 reports that underwent a screening process for the title and abstract for enhanced filtering. Of those, 390 were excluded, 197 were included in the final analysis, and 364 were subjected to a comprehensive report screening as their titles and abstracts were inconclusive. Of the latter, 250 articles were rejected as they were unrelated to object detection; the remaining were added to the final inclusion list. This resulted in a total of 311 articles pertaining to the topic of interest. Those 311 papers are the object of quantitative analysis. From those, papers with an annual citation rate lower than five (n = 231) were removed, and the remaining (n = 80) were further analyzed following a qualitative approach.

The tools used during this analysis were VosViewer [14] version 1.6.18, ScientoPy [15] version 2.1.0, with additional exploration carried out using Python. The application of these advanced analytical tools enabled a thorough examination of the literature and provided valuable insights into the field's current state.

The bibliometric analysis is a valuable procedure to gather the overall state-of-the-art of a specific field of research, allowing the reader to obtain information concisely and identify relevant patterns and trends for further research in the field [16]. VosViewer is a software tool able to create and explore maps based on network data [17], being able to analyze efficiently large amounts of text data, as is the case of scientific texts and academic literature [18]. On the other hand, ScientoPy is a scientometric tool capable of identifying topic trends efficiently, being able to manage and process datasets from the two main bibliographic databases, which are used in this study, namely Clarivate Web of Science (WoS) and Scopus [19].

## 3 Results

### 3.1 Quantitative analysis

In this section, a quantitative analysis was undertaken on 311 documents. This analysis was developed into five distinct perspectives, including an annual publication analysis, research area analysis, journal publication analysis, country publication analysis, author-publication analysis, and keyword analysis.

#### 3.1.1 Annual publication analysis

According to the data available in WoS and Scopus, 311 publications focused on object detection in medical imaging.

**Fig. 2:**
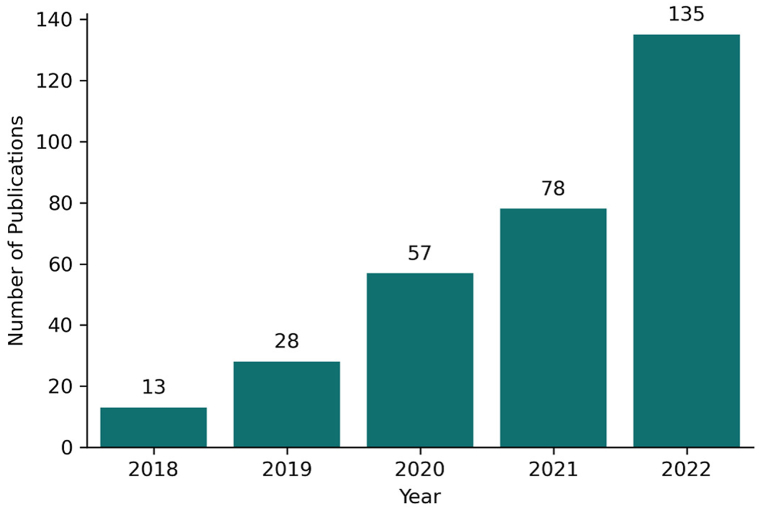
Growth of literature related to object detection in medical imaging until 2022.

The number of articles on the subject is the most basic indicator of publications trends. Fig. 2 shows that the first publications in the field date from 2018, with a consistent upward trend in the number of publications thereafter.

In 2018, only thirteen papers were published in the area. In 2019, more than twice as many articles as the previous year were published, and in 2022 the number of publications was around ten times higher than the first year. This change reflects the growing acceptance of this topic in the research community.

The citations of these 311 publications were analyzed as a whole, as shown in Table 1. In total, those articles were cited 5052 times, and only 5.79 % (n = 18) of the publications have more than 50 citations. On the other hand, 77.81 % (n = 242) of the total publications have at least one citation.

**Table 1:** Article papers and citations per year of object detection in medical imaging field.^a^

Year	TP	TC	AC	Total publications with citations				
				≥50	≥20	≥10	≥5	≥1
2018	13	1062	81.69	6	9	12	13	13
2019	28	799	28.54	6	14	22	23	27
2020	57	2358	41.37	6	18	36	47	57
2021	78	593	7.60	0	7	19	42	73
2022	135	240	1.78	0	0	2	14	72
%	100.00 %			5,79 %	15.43 %	29.26 %	44.69 %	77.81 %

a TP stands for total papers, TC for total citations, and AC for average article citations. An analysis of the number of publications according to their quantity of citations (higher or equal to 50,20,10,5 and 1) is performed for each year.

With the emergence of this field in 2018, the total number of publications in that year was significantly low. However, the average number of citations for those articles stands at a significant value of around 82 citations per publication. Despite a considerable decrease to an average of 28 citations per publication in 2019, the publications in 2020 achieved a peak of 41 average citations per year. This, combined with the increase in the number of publications in recent years, demonstrates the potential growth in this field.

#### 3.1.2 Research area analysis

**Fig. 3:**
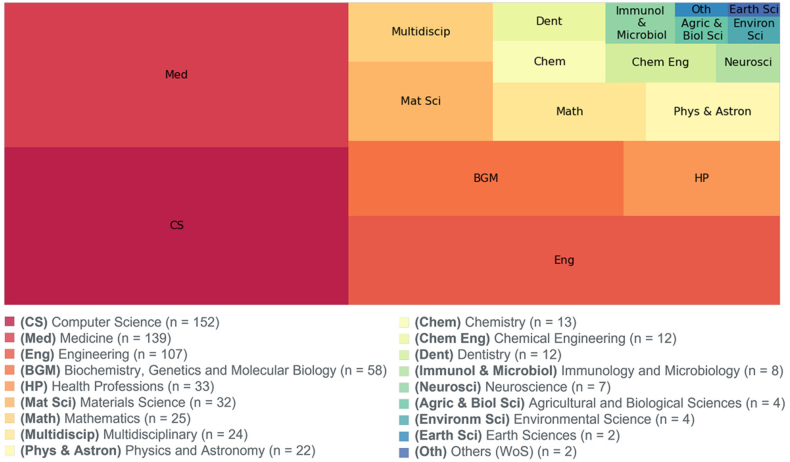
Treemap with the distribution of publications across various research areas provided by Scopus.

According to Scopus, and based on their subject area categories and classifications [20], the selected publications are associated with 17 distinct fields, where publications can be included in more than one area. In total, 656 links have been established between the 311 publications and the 17 research areas. As expressed in Fig. 3, the treemap clearly illustrates that most of the publications are included in Computer Science, Medicine, and Engineering, with 152, 139, and 107 records, respectively.

When viewed from a broader medical perspective, the areas of "Medicine" (n = 139), "Biochemistry, Genetics and Molecular Biology" (n = 58), "Health professions" (n = 33), "Dentistry" (n = 12), "Neuroscience" (n = 7) and "Immunology and Microbiology" (n = 8) correspond to approximately 39,30 % of the total associations.

From an engineering-oriented perspective, "Computer Science" (n = 152) and "Engineering" (n = 107) account for 39,60 % of the total links.

In a multidisciplinary view, publications from "Materials Science" (n = 32), "Multidisciplinary" (n = 24), "Mathematics" (n = 25), "Physics and Astronomy" (n = 22), "Chemistry" (n = 13), "Chemical Engineering" (n = 12), "Agricultural and Biological Sciences" (n = 4), "Environmental Sciences" (n = 4), account for around 20,80 % of the total number of links. Two publications were not available on Scopus and were classified as "Others".

#### 3.1.3 Source analysis

The 311 publications obtained were published in 161 journals from different fields of study. There were 111 journals (68.94 %) with only one publication associated, followed by 17 journals (10.60 %) with two articles published. Notably, only two journals have more than ten publications, namely IEEE Access (n = 17) and Scientific Reports (n = 13). All journals with five or more publications, as shown in Supplementary file 2, account for 33.12 % of the total publications and belong to the first quartile, excluding Diagnostics and Applied Sciences (Switzerland) Journals, which belong to the second quartile.

Among these journals, those with the highest average number of citations based on the total number of publications were "Computers in Biology and Medicine", with an average of 159 citations; Scientific Reports, with approximately 53 citations on average; and PLoS One, with an average of 24 citations.

#### 3.1.4 Countries publication analysis

In this analysis, each publication has been linked to the countries with which each author is associated. Since each publication can have more than one author, and an author can be associated with more than one country, a paper can be associated with one or further countries. However, if a paper has two authors from the same country, we only considered it once to avoid duplicating the same article in the same country.

At the time of research, 51 countries have published at least one paper. Table 2 shows countries with more than one hundred citations sorted by the number of citations.

**Table 2:** Countries with a higher number of citations.^a^

Rank	Country	P	C	AC
1	Japan	36	1791	49.75
2	United Kingdom	12	1465	122.08
3	Turkey	14	1451	103.64
4	Taiwan	17	1404	82.59
5	Singapore	2	1328	664.00
6	China	124	1273	10.27
7	United States	48	828	17.25
8	Hungary	1	364	364.00
9	South Korea	22	356	16.18
10	Australia	10	119	11.90
11	Germany	9	105	11.67
12	Hong Kong	6	116	19.33

a P stands for the number of publications, C for the number of Citations, AC is the average number of citations per publication.

Japan stands as the country with more citations (c = 1791) associated with its publications, followed by the United Kingdom (c = 1465) and Turkey (c = 1451). However, regarding the average number of citations, Singapore is at the front with 664 citations per publication, Hungary in second with 364, and the United Kingdom stands in third place with around 122 publications.

**Fig. 4:**
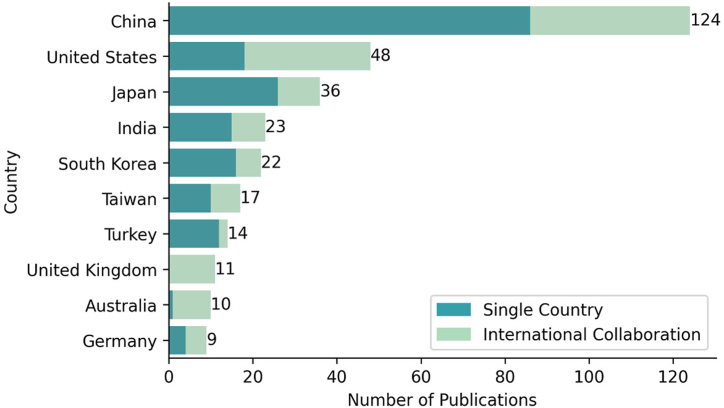
The top ten most productive countries and their international co-authorship.

Fig. 4 illustrates the top ten most productive countries, with a supplementary analysis of co-authorship, which indicates collaboration among authors from different countries. China is the leading country in this field, with 124 publications, followed by the United States with 48 publications and Japan with 36 publications. The United Kingdom displays the highest percentage of affiliations in terms of international collaboration, with all of its articles resulting from international partnerships. In contrast, Australia has only one out of ten papers with international collaboration. In absolute terms, China has published 86 papers with international collaboration out of 124 total papers, the United States has published 30 out of 48 papers with international affiliations, and Japan has published 26 papers out of 36.

When we assess the average number of citations of these three leaders in terms of publications, China presents a value of 10.27, the United States has 17.25, and Japan has 49.75, representing the 25th place, 15th place, and 7th place, respectively, among the 51 countries available.

#### 3.1.5 Authors publication analysis

In the set of 311 publications chosen, the author analysis was done through the SCOPUS ID of the authors. This approach was adopted to prevent the association of papers for authors with the same name, even if they are not the same researcher.

From this analysis, it was found that there were a total of 1870 related authors. Of those, 1752 authors were associated with only one paper, while only eight are associated with four or more papers, as seen in Table 3 (which lists the most productive authors in the field).

**Table 3:** The most productive authors.^a^

Rank	Author	Country	P	C
1	Ariji, Eiichiro	Japan	7	195
2	Aniji, Yoshiko	Japan	7	195
3	Katsumata, Akitoshi	Japan	6	182
4	Fukuda, Motoki	Japan	5	165
5	Liang, Yixiong	China	5	81
6	Fujita, Hiroshi	Japan	5	176
7	Muramatsu, Chisako	Japan	4	118
8	Kuwada, Chiaki	Japan	4	107

a Authors with four or more publications in the field. P stands for the number of papers, and C for the number of citations on those papers.

Of the 1870 related authors, nine have three publications, 100 have two publications, and the remaining have just one single publication. Notably, all the most productive authors (authors with four or more publications) belong to Japan or China.

With regard to the number of citations, out of all the authors, 32 are cited more than 100 times, 15 have received more than 200 citations, and 401 authors have no citation associated with their work. Table 4 reveals the identities of all authors with more than 200 citations.

**Table 4:** Authors with more than two hundred citations associated.^a^

Rank	Author	Country	P	C
1	Baloglu, Ulas Baran	United Kingdom	1	1306
2	Ozturk, Tulin	Turkey	1	1306
3	Rajendra Acharya U.	Singapore, Taiwan, Japan	1	1306
4	Talo, Muhammed	Turkey	1	1306
5	Yildirim, Eylul Azra	Turkey	1	1306
6	Yildirim, Ozal	Turkey	1	1306
7	Csabai, István	Hungary	1	364
8	Horváth, Anna	Hungary	1	364
9	Pollnar, Péter	Hungary	1	364
10	Ribli, Dezso	Hungary	1	364
11	Unger, Zsuzsa	Hungary	1	364
12	Lu, Le	United States	3	242
13	Yan, Ke	United States	3	240
14	Wang, Xiaosong	United States	1	228
15	Summers, Ronald M.	United States	1	228

a The authors with more than 200 citations are reported with their country, the number of publications associated to them, and the total number of citations.

As shown in Table 4, 14 out of the 15 authors with 200 or more citations associated have published only one paper on the field, and only two are associated with the publication of three papers, namely Le Lu and Ke Yan from the United States.

#### 3.1.6 Keyword analysis

**Fig. 5:**
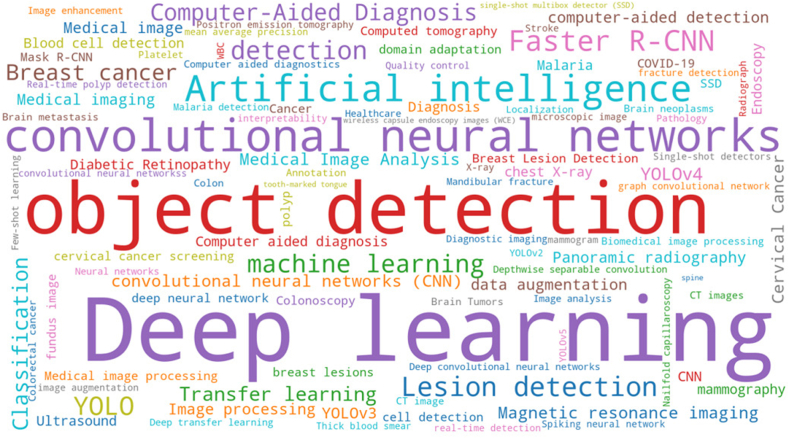
Wordcloud with keyword analysis. The size of a word or concept corresponds to the frequency of occurrence.

To gain insight into the central focus of the research, the commonly utilized words in the keyword section of the 311 selected articles were also evaluated. The analysis was conducted using the software ScientoPy. The word cloud in Fig. 5 illustrates the most frequently employed terms of the 311 selected articles. It is evident, as their frequency determines the size of the concepts in a word cloud, that concepts such as deep learning (n = 138), object detection (n = 84), convolutional neural networks (n = 54), and artificial intelligence (n = 34) are among the most frequently used terms.

Concerning the algorithms used, Faster R-CNN appears in 19 articles as a keyword, and the YOLO algorithm (regardless of the version) is present as a keyword in 31 records.

**Fig. 6:**
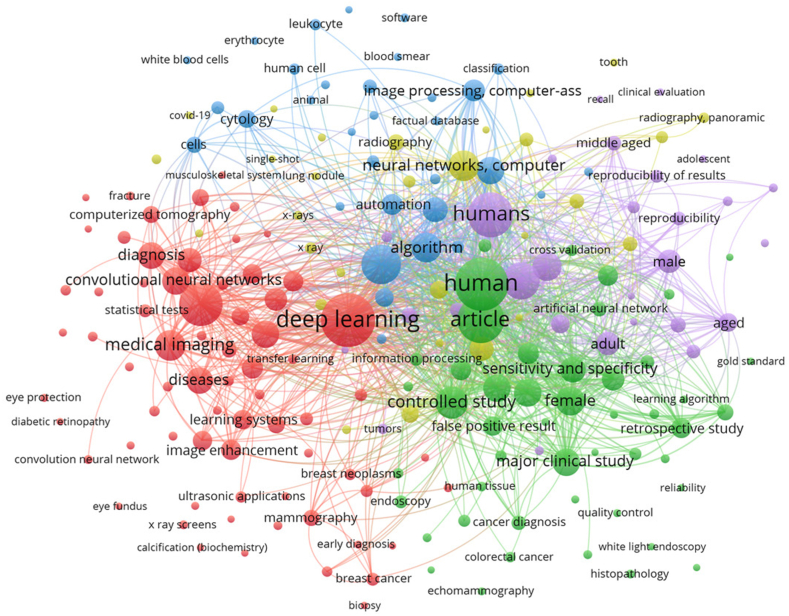
Co-occurrence of index keywords network developed in VosViewer. Only keywords with five or more occurrences are shown.

In Fig. 6, we report the network of co-occurrence of index keywords provided by VosViewer. In all the 311 articles, we defined the threshold where only keywords with five or more occurrences are shown. From the several keywords represented, Deep Learning appears 181 times, object detection has 123 appearances, and convolutional neural network appears 101 times.

Each node in Fig. 6 represents a keyword, and its size indicates the rate of frequency of the corresponding keyword, where the larger the node, the higher the significance of the keyword. The distance between two nodes reflects the strength of the relationship between two keywords. In other words, the closer two nodes are to each other, the higher the frequency of those two keywords appearing together.

Additionally, the color of the nodes defines the cluster to which each node belongs. VosViewer's clustering approach represents the connections and relations among nodes based on distance and density. The algorithm tries to identify sets of keywords that are prone to co-occur more frequently with each other than with keywords outside the cluster. To ensure visual singularity, each cluster is assigned a unique color.

From the network, we can verify four main clusters created. The red cluster seems to include articles where the main focus is on the technology used, with deep learning, medical imaging, and convolutional neural networks as the terms with more weight. There is a similar interpretation for the blue cluster with algorithm, neural network, and automation as the most significant keywords. The green cluster seems to point out articles more medically oriented, with human, article, and controlled study as the main keywords. The purple cluster has some similarities with the former cluster, with human, male, and aged as keywords with higher occurrence.

### 3.2 Qualitative analysis

The qualitative analysis was based on publications with an annual citation rate greater than 5. This selection resulted in 80 publications, with 30 presenting an annual citation rate greater than 10, as shown in Supplementary File 3.

In the papers undergoing a qualitative analysis, it is crucial to discern the incidence of algorithms used, the medical imaging modality most frequently associated with publications concerning object detection, and the anatomical application areas where object detectors are applied. These topics are thoroughly reviewed in the following three subsections.

#### 3.2.1 Used algorithms

**Fig. 7:**
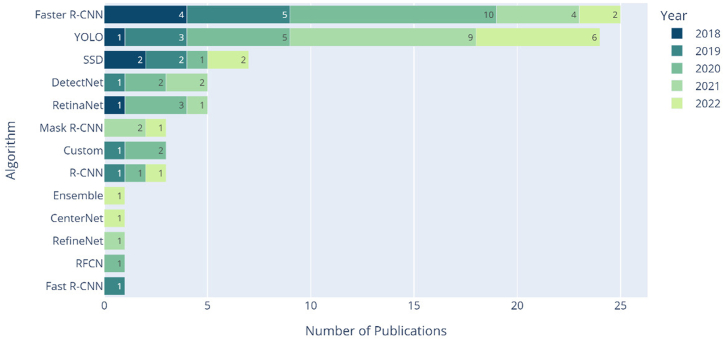
Stacked bar plot of the algorithms explored by year.

Concerning the object detection algorithms applied in the 311 publications, and as illustrated in Fig. 7, several algorithms have been applied as strategies to identify objects of interest in the given images. Among the most common, we should mention Faster R-CNN, YOLO, SSD, DetectNet, RetinaNet, Mask R-CNN, and R-CNN.

R-CNN, proposed in 2014 [21], was one of the first emerging techniques for object detection using convolutional neural networks. As a two-stage object detection algorithm, R-CNN has a first stage where regions of interest are proposed, while the second phase is focused on detecting the final boxes and classifying them.

The former algorithm's inefficiency was recognized due to its limitations on speed and accuracy. Faster R-CNN is proposed by Ren et al. [22] as an advancement to R-CNN, where, in the first stage, a Region Proposal Network (RPN) is implemented to generate more efficiently the proposals and, in the second phase, Fast R-CNN [23] is implemented to detect the final boxes and classify them. The RPN and the object detection network are trained simultaneously, allowing to reduce the computational cost, increase the training speed, and improve the detection.

Mask R-CNN [24] extends the Faster R-CNN approach by adding the capability to perform instance segmentation at the pixel level to the task of detecting objects of interest.

One-stage detectors emerged with Single Shot MultiBox Detector (SSD) [25] and You Only Look Once (YOLO) [26]. These detectors possess a simpler architecture than two-stage detectors and simultaneously locate and classify each object without the need for a region proposal process. The main difference between these two approaches is that the former computes a feature map and combines distinct grids of different sizes to better detect objects of any size. The latter, on the other hand, is faster by defining one single grid where class probabilities are associated with anchor boxes with different scales and aspect ratios.

In posterior years, other one-stage approaches appeared, such as RetinaNet [27] and DetectNet [28]. RetinaNet introduces the concept of Focal Loss to the classification where different weights are assigned to hard samples, solving the problem of class imbalance and improving the detection performance. DetectNet is an object detection architecture created by NVIDIA and optimized for real-time object detection.

While two-stage detectors, at the emergence of object detection architectures, tended to yield higher detection accuracy, but also a slower training phase [29], with the advance and development of one-stage frameworks, the performance of both types appears to be similar, if not better in the latter one.

Faster R-CNN stands as the most frequently used algorithm, with 32.5 % of the publications, followed by the YOLO algorithm (independently of the version) with 30 % of the records, and SSD with 8.75 %. RetinaNet and DetectNet have five publications associated, and the remaining 13 papers (16.25 %) apply other object detectors, as shown in Fig. 7.

Faster R-CNN was the most widely utilized algorithm during the emergence of object detection in medical imaging. However, since 2019, the algorithm YOLO has gained significance in the field, with more publications in 2021 and 2022 than Faster R-CNN, which was previously the most popular algorithm. This fact reveals that the YOLO algorithm is gradually replacing the position previously held by Faster R-CNN and has gained increased popularity in recent years.

#### 3.2.2 Modalities

**Fig. 8:**
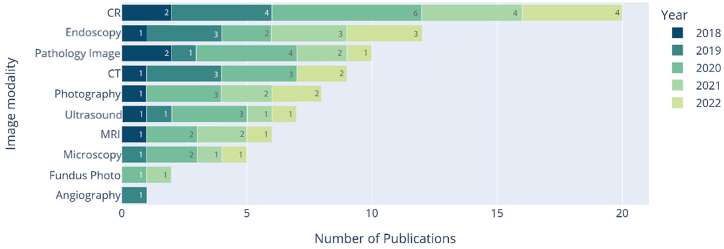
Stacked barplot of the used image modalities by year.

There are several types of medical imaging modalities. As shown in Fig. 8, the most frequent modalities include Computed Radiography (CR), Pathological Imaging, Endoscopy, and Computed Tomography (CT).

Computed Radiography is a widely accessible and affordable screening method broadly used to detect several pathologies, such as lung diseases and mass screening. As an example, this method is applied to mammography in a low-dose X-ray approach to visualize the breast structure and identify possible breast anomalies [30].

Endoscopy allows the visual inspection of an internal organ or tissue in detail [30]. One possible application of endoscopic procedures is to identify colorectal lesions, where techniques such as high-resolution microendoscopy, fluorescence imaging, and enhanced endoscopy are available to improve the detection rate of tumors using endoscopy [31].

Pathology Imaging uses static images, live streaming of images, and whole slide imaging (WSI) to make pathology diagnoses. The latter allows the digitalization of entire glass slides [32],enabling quantitative analyses for the entire landscape of tissue morphology without the need for microscopy [33].

Digital photography plays an important role in various specific medical specialties, enabling the identification and diagnosis of potential lesions and diseases in a cost-effective, noninvasive, readily accessible, and real-time manner. The usage of this approach encompasses a wide range of tasks, including the detection of skin cancer, dermoscopy imaging, assessment of oral and dental health, and the diagnosis of hair and nail conditions [34].

Ultrasound is a widely used medical imaging modality, providing a noninvasive technique for imaging human anatomy [35], being widely applied in prenatal screening due to its relative safety, low cost, noninvasive nature, and real-time display [36].

Magnetic Resonance Imaging (MRI) is a noninvasive method for acquiring high-resolution images of any organ in the body. This imaging technique can be applied to different tasks, such as stroke detection, tumorigenesis, and myocardial infarction [37].

Microscopy images can support quantitative analysis for diagnosing and characterization of several diseases, playing an essential role in computer-aided diagnosis and prognosis [38]. Associated with Histopathology, the field of study of human tissue using a microscope, it allows, through staining, the visualization of specific parts of tissues that enables the identification of different diseases such as kidney cancer, lung cancer, and breast cancer, among others, through nuclei and cell detection [30].

Fundus Photography is a non-contact ophthalmologic examination of the eye, considered the most cost-effective imaging modality for screening fundus disease, that allows the visualization of several eye lesions such as of the optic nerve and retina [39].

Conventional angiography and contrast-enhanced magnetic resonance angiography is regarded as the reference examination for investigation of atherosclerotic lesions of the supra-aortic extracranial vessels, and especially for detecting stenosis of the carotid artery, being the latter one the less invasive and with similar results to the former [40].

Of the 80 papers analyzed, 25 % utilized CR as the primary image modality, followed closely by Endoscopy with 15 % and pathology imaging with 12.50 % of the publications. Although it is unclear whether there have been significant changes in the type of images used over the years, it is evident an increase in the use of pathological images in 2021 and 2022.

#### 3.2.3 Anatomical application areas

**Fig. 9:**
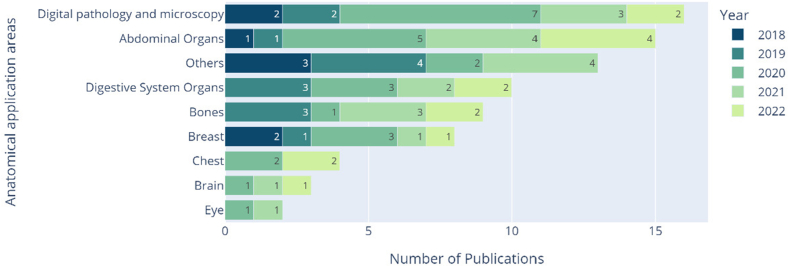
Stacked barplot of the anatomical application areas explored by year.

Over the past five years, the anatomical application areas where object detection has been applied have been diverse, but digital pathology and microscopy have emerged as the most popular, with a total of 16 publications, as illustrated in Fig. 9. Closely following in popularity is the detection of abnormalities in abdominal organs, with 15 publications.

In the following subsections, we will qualitatively analyze the various publications distributed by their specific anatomical application area.

##### 3.2.3.1 Abdominal organs

As shown in Table 5, on abdominal organs, the research is focused on the colon, gallbladder, liver, stomach, cervix, and uterus.

**Table 5:** Publications in which object detection algorithms are applied to abdominal organ images.^a^

Reference	C	Modality	Organ	Algorithm-based	Task
[41]	98	E	Colon	YOLO	Polyp detection
[42]	52	E	Colon	SSD	Polyp detection
[43]	16	E	Colon	YOLO	Polyp detection
[44]	7	E	Colon	SSD	Polyp detection
[45]	14	E	Colon	RefineNet	Polyp detection
[46]	21	E	Gallbladder	YOLOv3	Detection of bile duct injury
[47]	41	CT	Gallbladder	YOLOv3	Detection of gall stones
[48]	17	US	Liver	Faster R-CNN	Abnormality detection
[49]	25	E	Stomach	Faster R-CNN	Detection of gastric lesions
[50]	9	E	Stomach	YOLOv3	Detection of gastric lesions and neoplasm prediction
[51]	13	PH	Cervix	R-CNN	Cervical cancer detection
[52]	27	PH	Cervix	Faster R-CNN	Cervical cancer detection
[53]	18	PH	Cervix	Faster R-CNN	Cervical cancer detection
[54]	30	US	Uterus	YOLOV2	Detection of cardiac structural abnormalities
[55]	27	US	Uterus	Custom	Detection of cardiac structural abnormalities

a Overview of papers using object detection techniques for detecting abnormalities in abdominal organs, where E stands for endoscopy, CT for computed tomography, PI for pathology image, PH for photography, and US for ultrasound. In the header of the table, C stands for the number of citations.

Five of the selected papers are dedicated to colon polyps detection, using images obtained through endoscopy. Among those publications, YOLO and Faster R-CNN are applied twice, while RefineNet is applied once. In the first study in which YOLO is implemented, an improved framework is developed to detect polyps in a dataset consisting of more than 17,000 frames, yielding a precision of 88.6 % and a recall of 71.6 % at a prediction speed of 6.5 frames per second [41]. In the second study, YOLOv3 and YOLOv4 architectures are applied to two datasets, achieving a precision of 90.6 % and a recall of 91.0 % at a processing speed of 100 frames per second [43].This last publication combines the YOLO structure with CSPNet to obtain a higher-performance model. In the SSD Implementations, one of the studies is validated in 7077 images, where the model was able to identify correctly 1073 colon polyps out of the existent 1172, with a sensitivity of 90 %, and a processing time of 20 frames per second [42]. In the second implementation, a multiscale pyramidal fusion single-shot multibox detector network is applied, resulting in an mAP of 93.4 % [56], with a testing speed of 62.5 FPS. RefineNet is also employed for polyp detection and applied to a combination of four public data sets. In this study [45], the authors compare RefineNet with different algorithms such as YOLO and Faster R-CNN, achieving the best performance among all the tested approaches with an mAP of 73.5 %, while YOLOv3 and SSD achieve the fastest inference with 60 FPS.

Two studies have been conducted using YOLOv3, with distinct objectives concerning gallbladder organs. In the first one, endoscopic images were used to detect four landmarks to prevent bile duct injury in 23 short videos. The authors of this study recognize the system's suboptimal performance [46]. In contrast, the second study uses 223,846 CT images to detect gall stones, achieving an mAP of 89.5 % for granular and muddy stones [47].

Regarding the liver, one study has been conducted in which cysts and primary hepatic carcinoma detection in ultrasound imaging were accomplished using Faster R-CNN with a ResNet backbone. In this publication [48], the authors achieved an mAP of 60 %, representing an improvement over the results obtained with YOLOv2, which had an mAP of 51 %.

In two different studies, the detection of gastric lesions on endoscopic imaging was approached. In the Faster R-CNN implementation [49], more than one million images were employed to train and test the system. The authors of this study enhance the significant difference in detection time between the automatic approach and clinicians’ performance. The results are reported by sensitivity values, ranging from 87.2 % to 99.3 %. In the second publication, where YOLOv3 was applied, the data was obtained from over 100,00 patients, and the final model sensitivity ranged between 91.7 % and 96.9 % [50].

Three of the fifteen papers on abdominal organs are focused on cervical cancer detection, obtained through applying two-stage object detection approaches on photographic images. In a study published in 2022 [57], a generative adversarial network was developed using an R-CNN to accurately detect small cervical cells and classify them into normal, precancerous, or cancerous. The remaining two publications employed Faster R-CNN. The first study created a system based on 2853 images labeled as high-risk or low-risk, achieving an AUC value of 0.87 [52]. In the second study, an improved version of Faster R-CNN was developed [53] and tested on 2528 images with 5294 lesion regions, achieving an mAP of 80.37 %. This represented better results than those obtained with SSD (mAP = 74.27 %), YOLOv3 (mAP = 77.11 %), and Faster R-CNN (mAP = 75.89 %).

Out of the fifteen papers identified, seven of them use one-stage object detectors.

Moreover, we assess additional relevant aspects, including the data sets employed, their magnitude, data accessibility, the integration of external organizational data, and the study's temporal orientation concerning being retrospective or prospective. The outcomes are summarized in Table 1 of Supplementary File 4.

##### 3.2.3.2 Bones

Nine of the eighty studies selected based on the annual citation rate are employed in bone imaging, as exposed in Table 6.

**Table 6:** Publications in which object detection algorithms are applied to bone images.^a^

Reference	C	Modality	Algorithm	Task
[58]	73	CT	Faster R-CNN	Detection of distal radius fractures
[59]	66	CR	DetectNet	Detection of mandible fractures
[60]	28	CR	DetectNet	Detection of maxillary sinus lesions
[61]	7	US	YOLOv3	Detection of vertebras in spine
[62]	7	CT	Ensemble	Detection of wrist fractures
[63]	13	CR	YOLOv3	Detection of hip dislocation
[64]	12	MRI	YOLOv3	Detection of Lumbar Disc Herniation
[65]	22	CR	Faster R-CNN	Skeletal bone age assessment
[66]	10	CT	YOLOv3	Detection of vertebral fractures

a Overview of papers using object detection techniques on bones, where CT stands for computed tomography, CR for computed radiography, US for ultrasound and MRI for magnetic resonance imaging. In the table's header, C stands for the number of citations.

On CT imaging, three studies use object detection to locate fractures, namely on the distal radius [58], on the wrist [62], and on the vertebras [66]. In the first one, a comparison is made between the automatic approach using a Faster R-CNN, and professional assessment. The authors conclude that the network achieved performances similar to those obtained from orthopedists and superior to radiologists. In a second study, wrist fractures are located with an Ensemble technique based on WBF. Ten base models are tried out, and the highest AP Score was obtained with the ensemble version obtained from the base learners. The authors enhance the potential of ensemble techniques in this field. In the third publication, vertebral fractures are detected using a YOLOv3 architecture, and one remark appointed by the authors is the highest interobserver reliability of the automatic system compared to human observers.

CR imaging is used in object detection studies with different purposes. Two publications, in particular, apply DetectNet [87], a network developed in DIGITS. The first study employs a model built on 210 training images to locate mandible fractures, achieving a sensitivity of 88 % in the testing data [59]. The second study, also utilizing DIGITS, identifies maxillary sinus lesions, with a sensitivity of 100 % on healthy and inflamed maxillary sinuses and 98 % and 89 % on distinct data sets for cyst maxillary sinus region [60]. Another study trains YOLOv3 to assess the risk of hip dislocation on 1490 radiographs from dislocated cases and 91,094 from non-dislocated cases, achieving a sensitivity of 89 % [63]. A Faster R-CNN is employed to assess skeletal bone age by detecting the ossification centers of the epiphysis and carpal bones [65], with a performance measured by MAE, achieving values of 0.48 and 0.51 in two tested data sets.

On ultrasound imaging, various object detectors, such as SSD, YOLOv3, and YOLOv4, were trained to detect the vertebrae in the spine [61]. While SSD achieved the highest mAP with a value of 90.84 %, YOLOv3 was chosen as the final model due to its short inference time of around 7 FPS, around ten times faster than SSD's.

An automatic detection system based on YOLOV3 and MRI was also proposed to assist in the initial lumbar disc herniation exam for lower back pain [64]. In this study, an mAP of 92.4 % was obtained at 550 images with data augmentation.

Table 2 of Supplementary File 4 summarizes the data sets used and the type of study applied to bone images.

##### 3.2.3.3 Brain

An MRI is a powerful tool for producing detailed images of the brain, which can be used to detect a wide range of conditions, such as tumors, injuries, strokes, and blood vessel problems. In recent years, some object detection algorithms have been applied to this field to automate the diagnostic process and increase the accuracy of results. Table 7 exposes those studies.

**Table 7:** Publications in which object detection algorithms are applied to brain images.^a^

Reference	C	Modality	Algorithm	Task
[67]	9	MRI	YOLOv5	Brain tumor diagnosis
[68]	20	MRI	YOLOv4	Brain tumor diagnosis
[69]	11	MRI	Faster R-CNN	Stroke lesion detection

a Overview of publications that employ object detection techniques on the brain, where MRI stands for magnetic resonance imaging. In the header of the table, C stands for the number of citations.

In the publications considered for qualitative analysis, two studies applied the YOLO architecture to brain tumor diagnosis, while another study applied Faster R-CNN for stroke lesion detection. In the study where YOLOv5 is applied [67], the authors compared the performance of different algorithm variants on 800 images to brain tumor detection. Unsurprisingly, the larger version of the model achieved the best mAP at 91.2 % using YOLOv5x, while the smaller versions of YOLOv5 (YOLOv5n and YOLOv5s) achieved the worst performances with an mAP of 85.2 % and 87 %. However, the trade-off is that larger versions take more time to train.

In a second study for brain tumor diagnosis and detection, a transfer learning approach and fine-tuning techniques were applied to YOLOv4 to build a model on a data set of 3064 MRI scans, which was able to detect three distinct tumors, namely glioma, meningioma, and pituitary. This improved version of YOLO achieved a final mAP of 93.14 % [68].

A third study on Brain MRI scans is focused on stroke lesion detection [69]. In this study, the authors compared the performance of Faster R-CNN, YOLOv3 and SSD, and concluded that SSD performed the best, with the highest mAP (89.77 %). When comparing the inference time, YOLO was the fastest approach, but this advantage was reflected in a significant decrease in the mAP, with a value of 74.9 %.

Table 3 of Supplementary File 4 provides a summary of the data sets employed and the study methodologies applied to brain images.

##### 3.2.3.4 Breast

Breast cancer is the most common cancer diagnosed worldwide, with 685,000 deaths associated in 2020, being estimated that in 2040 this value will rise to 1 million [70]. With eight publications associated, as expressed in Table 8, object detectors in breast imaging focus on detecting lesions.

**Table 8:** Publications in which object detection algorithms are applied in breast images.^a^

Reference	C	Modality	Algorithm	Task
[71]	364	CR	Faster R-CNN	Breast lesion detection
[72]	90	CR	YOLO 9000	Breast lesion detection
[73]	78	US	SSD	Breast lesion detection
[74]	43	CR	Custom	Breast lesion detection
[75]	61	CR	RetinaNet	Breast lesion detection
[76]	21	MRI	RetinaNet	Breast lesion detection
[77]	6	CR	Mask R-CNN	Breast lesion detection
[78]	10	CR	YOLOv3	Breast lesion detection

a Overview of publications using object detection techniques for breast lesion detection, where CR stands for computed radiography, US for ultrasound and MRI for magnetic resonance imaging. In the header of the table, C stands for the number of citations.

Various object detection models are applied in these studies, including YOLO, SSD, RetinaNet, Faster R-CNN, Mask R-CNN, and custom-designed networks. The most commonly used imaging modality was CR, but ultrasound images and MRI were also used.

Regarding CR imaging, six studies were conducted using different algorithms as a basis. One of the publications [71] applied Faster R-CNN to detect and classify malignant or benign lesions on a mammogram in a data set with 2620 screening exams. The system achieved an AUC of 0.85, with 10 % of the malignant lesions being missed. RetinaNet was tested in seven different data sets in the same year and, according to the authors, outperformed the conventional mass detection models [75]. In 2020, two publications addressed this task. One of these applied YOLO 9000 on two distinct data sets, comprising 600 and 360 images, respectively. 99.17 % of the total cases were predicted correctly on these data sets, with 0.83 % being false detections [72]. In the second case, a custom network was built to address class imbalance for small lesion detection on breast [74]. Another publication used a YOLO-based model on 235 publicly available mammograms and 487 privately collected mammograms, achieving a detection accuracy rate of more than 95 % for the distinct datasets used and an inference time of around 0.55 s per image [78]. In 2022, a Mask R-CNN-based framework was developed and implemented on public and in-house datasets. The model's performance was analyzed only using recall, achieving values of 0.82 for the first data set and 0.87 for the private data set, with an IoU threshold of 0.5 [77].

In 2019, ultrasound images were applied on distinct object detectors, including Fast R-CNN, Faster R-CNN, YOLO, YOLOv3 and SSD. YOLO, and SSD performed significantly better than the other methods, and SSD was chosen as the most suitable model for this task, achieving an mAP of 96.89 % in a total of 1041 cases [73].

Regarding MRI imaging, only one publication was included in this qualitative analysis [76], using RetinaNet. The authors compared the sensitivity, specificity, and AUC between the AI system and human readers, concluding that the former had a better diagnostic performance.

Table 4 of Supplementary File 4 summarizes the data sets used and the study methodologies applied to breast images.

##### 3.2.3.5 Chest

In thoracic image analysis of both computed radiography and tomography, the detection of pulmonary tuberculosis and COVID-19 were the most commonly addressed applications, as seen in Table 9.

**Table 9:** Publications in which object detection algorithms are applied in chest images.^a^

Reference	C	Modality	Algorithm	Task
[79]	1306	CR	YOLO 9000	COVID-19 detection
[80]	9	CT	CenterNet	Pulmonary tuberculosis detection
[81]	7	CR	SSD	COVID-19 detection
[82]	19	CR	Faster R-CNN	Pulmonary tuberculosis detection

a Overview of publications that apply object detection techniques on chest imaging for COVID and pulmonary tuberculosis detection, where CT stands for computer tomography and CR for computed radiography. In the table's header, C stands for the number of citations.

SSD and YOLO 9000 are applied to COVID detection on CR imaging. Several backbones have been tested with SSD, including VGG, Residual Network, DarkNet, and DenseNet, and their performance has been compared. DenseNet achieves the highest performance, with a precision of 0.93 and a recall of 0.94 [81]. The most cited publication in our qualitative research focuses on implementing YOLO for COVID detection. The proposed model is developed to provide accurate diagnostics for binary classification (COVID vs No-findings) and multi-class classification, where Pneumonia is added to the former classes. The authors report an accuracy of 98.08 % on the binary problem and 87.02 % for the multi-class problem [79].

In pulmonary tuberculosis detection, a Faster R-CNN adapted model has been applied to a multi-class problem on CT images, which encompasses co-existing cases of exudation, calcification, nodules, miliary tuberculosis, and other related conditions. This model achieves an mAP of 53.74 %, outperforming the original Faster R-CNN model, which reached 22.66 % mAP and surpassing the results obtained with FPN, which achieved 50.96 % mAP [82]. Additionally, CenterNet has also been used on 892 CT scans for pulmonary tuberculosis detection, yielding an mAP of 68 % [80].

The data presented in Table 5 of Supplementary File 4 defines the data sets employed and the research methodologies specific to the analysis of chest images.

##### 3.2.3.6 Digestive system organs

Concerning the digestive system, object detection techniques have been applied to various forms of medical imaging, including esophagus imaging obtained through endoscopy, tongue images obtained through photography, and teeth images obtained through CR imaging, as seen in Table 10.

**Table 10:** Publications in which object detection algorithms are applied to images of digestive system organs.^a^

Reference	C	Modality	Organ	Algorithm	Task
[83]	92	E	Esophagus	YOLOV2	Early esophageal neoplasia detection
[84]	44	E	Esophagus	SSD	Early esophageal adenocarcinoma detection
[85]	29	PH	Tongue	R-CNN	Tooth-marked tongue detection
[86]	117	CR	Teeth	Faster R-CNN	Tooth detection
[87]	58	CR	Teeth	DetectNet	Root fracture detection
[88]	24	CR	Teeth	DetectNet	Tooth detection
[89]	26	CR	Teeth	Faster R-CNN	Periodontal compromised teeth detection
[90]	8	CR	Teeth	Faster R-CNN	Permanent teeth detection
[91]	11	CR	Teeth	Faster R-CNN	Dental disease detection
[92]	5	CR	Teeth	Faster R-CNN	Marginal bone loss around implants detection

a Overview of publications that apply object detection techniques on digestive system organs, where CR stands for computed radiography, E for endoscopy and PH for photography. In the header of the table, C stands for the number of citations.

In particular, YOLOv2 and SSD have been applied to endoscopy imaging, with the former being used to detect early stages of esophageal neoplasia [83] and the latter being used to detect early stages of esophageal adenocarcinoma [84]. In the first study, 916 images were used to train the model, and 458 images were used to test it, achieving an mAP of 75.33 % for narrow bone imaging and 80.19 % for near-focus images at a speed of 45 FPS. In the second study, the performance of SSD and Faster R-CNN were compared in terms of average recall rate (ARR), average precision rate (APR), sensitivity (SE), specificity (SP), and F-measure (FM) using a 5-fold cross-validation method. SSD achieved the highest values with 0.7 on APR, 0.90 in SE, 0.88 in SP, and 0.88 in FM, being surpassed by Faster R-CNN only in ARR with a difference of 0.04.

In a tongue photography study, the goal was to identify tooth-marked areas on the tongue using a data set containing 641 images, 297 of which were tooth-marked. An adapted version of R-CNN was applied, and the authors reported an average accuracy of 72.7 %, a TPR of 69.1 %, and a TNR of 76.2 % [85].

Tooth detection appears to be a prevalent subject in the realm of object detection frameworks in medical imaging. Seven of the 80 studies analyzed were dedicated to tooth detection and related tasks, and the Faster R-CNN algorithm was applied in five of these studies. In the first study [86], the model achieved a precision and recall rate of over 90 %, with a mean average precision (mAP) of 91 %. These values were only attainable after implementing several postprocessing procedures.

In another study, Faster R-CNN was employed for Periodontal compromised teeth detection [89], resulting in an accuracy rate of 80 %, a positive predictive rate of 81 %, a sensitivity of 84 %, a specificity of 88 %, and a F-measure of 81 %. The authors of this study concluded that the system exhibited satisfactory detection abilities.

Faster R-CNN was also applied to permanent teeth detection in a study [90] where the authors reported results of 0.99 for recall and precision. Additionally, the algorithm was used in another work [91] to detect dental diseases such as decay, periapical periodontitis, and periodontitis at varying levels of severity. The results showed that decay and periapical periodontitis lesions were detected with precision, recall, and average precision values of less than 0.25 for mild levels, while moderate and severe levels had values ranging from 0.2 to 0.3 and 0.5–0.6, respectively.

In the more recent study where Faster R-CNN was applied, the goal was to detect Marginal bone loss around dental implants [92]. The system was evaluated using a dataset of 1670 images and was assessed in terms of sensitivity, specificity, diagnostic error rate, omission diagnostic rate, and positive predictive value. Kappa statistics were also compared between the system and dental clinicians, and the authors concluded that there was a high level of agreement between the automatic system and the clinicians.

On DetectNet, two studies are implemented, both applied to CR imaging. In one of these studies, the primary objective was the detection of root fractures [87]. The study employed 300 images, containing a total of 330 root fractures. Out of these, 267 were successfully detected by the automatic approach, while twenty were falsely detected, resulting in a recall of 0.75, a precision of 0.93, and an F measure of 0.83. In a second study [88], DetectNet was applied to tooth detection. However, the authors acknowledged several limitations in their study, such as the small data set used, which was composed of only 75 training cases and 25 test cases.

Table 6 of Supplementary File 4 summarizes the data sets used and the type of study applied to images of digestive system organs.

##### 3.2.3.7 Digital pathology and microscopy

The growing availability of large-scale gigapixel whole-slide images of tissue specimens has made digital pathology and microscopy a highly popular application area for deep learning techniques [93]. These imaging modalities have been applied to various purposes, such as cell detection and malaria detection, among others, as seen in Table 11.

**Table 11:** Publications in which object detection algorithms are applied to digital pathology and microscopy images.^a^

Reference	C	Modality	Organ	Algorithm	Task
[94]	93	PI	Blood	Faster R-CNN	White blood cells detection
[95]	37	PI	Tissue	Faster R-CNN	Glomerular detection in multistained human renal tissue
[96]	27	M	Others	Faster R-CNN	Human intestinal organoid detection
[97]	16	M	DNA	Faster R-CNN	Detection of DNA damage
[98]	29	PI	Cervical cells	YOLOv3	Cervical cell recognition
[99]	31	M	Blood	YOLO	Malaria detection
[100]	73	PI	Blood	SSD	Peripheral leukocyte recognition
[101]	53	PI	Blood	YOLO	Blood cells detection
[102]	18	PI	Blood	YOLOv4	Blast cell detection
[103]	15	PI	Blood	RetinaNet	Malaria detection
[104]	9	PI	Brain tissue	YOLOv3	Alzheimer's diagnose
[105]	22	PI	Blood	R-CNN	Cells detection
[106]	15	M	Others	Faster R-CNN	Detection of cellular organelles
[107]	12	PI	Liver and Kidney tissue	RetinaNet	Diatom detection
[108]	24	M	Others	RetinaNet	Pulmonary Hemosiderophages detection
[109]	8	M	Others	YOLOv4	Sperm detection

a Overview of publications that apply object detection techniques on digital pathology and microscopy imaging, where PI stands for pathological imaging and M for microscopy imaging. In the header of the table, C stands for the number of citations.

The YOLO algorithm is regarded as the most prevalent among these studies, with six out of sixteen publications devoted to its application. In particular, three studies have explored the use of YOLO in the analysis of blood samples, specifically for detecting blood cells [101], blast cells [102], and malaria [99]. In the first study, YOLO was trained and tested using 364 images with red blood cells, white blood cells, and platelets. The results showed an mAP of 82.58 % using the VGG16 backbone. Another study [94] compared YOLOv3 with other popular models, including R-CNN, Fast R-CNN, Faster R-CNN, and SSD. Ultimately, the Faster R-CNN model arose as the winner with an mAP of 71 % and an inference time of 609 ms per image with VGG16. A third study on blood smears applied 14,700 images of peripheral leukocytes with eleven distinct categories, resulting in an mAP of 93.10 % for SSD and an inference time of 53 ms per image. Although YOLOv3 also performed well, it achieved only 92.10 % mAP. The blast cell detection study [102] applied YOLOv4 on blood smears to aid in early leukaemia diagnosis and achieved an mAP of 95.57 % with an inference value of 50 FPS. In the malaria detection study with microscopic images, YOLOv3 and YOLOv4 were compared, with the last being the superior model, achieving an mAP of 96.32 % and an inference rate of 29.60 frames per second. Another study [108] applied RetinaNet to detect malaria in 169 microscopic images for train and 130 for validation, with results reported in terms of sensitivity (0.92), specificity (0.90), accuracy (0.91), positive predicted value (0.92) and negative predicted value (0.90).

The YOLO object detector has also been used for cervical cell recognition [98] and sperm detection [109]. In the first study, YOLOv3 was applied to cervical cytology screening and achieved an mAP of 63.4 %, with 95.7 % of sensitivity and 67.8 % of specificity, on a data set of 12,909 images split into ten distinct categories.

In the sperm study, with 125,000 objects annotated, YOLOv4 and other models such as YOLOv3, SSD, RetinaNet, and Faster R-CNN were compared, with YOLOv4 and SSD achieving the highest mAPs of 40.50 % and 41.98 %, respectively. However, YOLOv4 was selected due to a higher AP on the class impurity.

The last study on YOLO was on Alzheimer's diagnosis [104]. The YOLOv3 was trained to detect five tau lesion types, including neuronal inclusions, neuritic plaques, tufted astrocytes, astrocytic plaques, and coiled bodies, on 2522 immunostained slides images of the motor cortex, achieving a maximum mAP of 74.4 %.

The Faster R-CNN algorithm was the second most used among the studies, appearing in five of the sixteen works available. In the study of glomerular detection in multistained human renal tissue [95], Faster R-CNN was used to automate the task and was trained on 33,000 images. The results were reported regarding recall, precision, and F measure for four different classes. Detection of human intestinal organoids [96] was performed using Faster R-CNN on a data set of 1750 image patches with 14,242 ground truths, resulting in an mAP of 80 %. The authors argue that the quality of the automatic detection was similar to human capability but substantially faster. Another study, examining DNA damage detection [97], reported an mAP of 74 % with Faster R-CNN, while SSD and YOLOv3 achieved mAPs of 22 % and 30 %, respectively. The detection of cellular organelles [106] was also considered using Faster R-CNN, with results reported solely for classification purposes.

The RetinaNet algorithm was used in three studies. Excluding the malaria study also reported in this subsection, this algorithm was used on liver and kidney tissue for diatom detection [107], and to detect Pulmonary Hemosiderophages [108]. The first study obtained an average precision of 0.82 and an average recall of 0.88. In the second study, a RetinaNet was trained on seventeen completely annotated cytology whole slide images (WSI) containing 78,047 hemosiderophages and achieved an mAP of 66 % for the five classes approached, exceeding human expert concordance.

The R-CNN algorithm was only applied in one of the studies for cell detection [105], with a dataset of 600 training images and 100 testing images, and achieved an mAP of 82 %.

Table 7 of Supplementary File 4 provides a summary of the data sets employed and the study methodologies applied to digital pathology and microscopy images.

##### 3.2.3.8 Eye

The field of ophthalmologic imaging has experienced significant growth in recent years, but it is only in recent times that deep learning techniques have been harnessed for eye image analysis, with a majority of the studies centering around color fundus imaging [93].

The qualitative analysis of the eighty studies revealed that only two of them have been applied to eye imaging, as expressed in Table 12.

**Table 12:** Publications in which object detection algorithms are applied to eye images.^a^

Reference	C	Modality	Algorithm	Task
[110]	29	FP	Faster R-CNN	Diabetes-Based Eye Disease Detection
[111]	12	FP	YOLOv3	Lesion detection

a Overview of publications that apply object detection techniques on eye imaging, where FP stands for fundus photography. In the header of the table, C stands for the number of citations.

The first study used FRCNN, a Keras implementation of Faster R-CNN, to identify diabetes-based eye diseases, including diabetic retinopathy, diabetic macular edema, and glaucoma [110]. The authors achieved an mAP of 94 % with the proposed model, outperforming two other approaches, SSPnet and R-CNN, which achieved mAPs of 85 % and 89 %, respectively. In the second study [111], the authors used YOLOv3 to identify lesions in eye images. The results were reported in terms of average precision (0.08 and 0.52), average recall (0.86 and 0.91), and F1 Score (0.16 and 0.66) for both the number of lesions and the number of images analyzed.

Table 8 of Supplementary File 4 outlines the data sets used and the type of study applied to eye images.

##### 3.2.3.9 Other anatomical application areas

This final subsection lists papers that address multiple applications, where the organ is not unique, and diverse applications that were not included in the previous subsections, as seen in Table 13.

**Table 13:** Publications in which object detection algorithms are applied to various topics.^a^

Reference	C	Modality	Organ	Algorithm	Task
[112]	38	PH	Foot	Faster R-CNN	Diabetic foot ulcers detection
[113]	37	PH	Skin	Mask R-CNN	Melanoma detection
[114]	19	PH	Skin	Faster R-CNN	Scalp health diagnosis
[115]	98	US	Thyroid	Faster R-CNN	Thyroid papillary cancer detection
[116]	27	A	Vessels	Custom	Intracranial aneurysm detection
[117]	10	CT	Lymph nodes	DetectNet	Cervical lymph nodes detection
[118]	228	CT	Several	Faster R-CNN	Lesion detection
[119]	54	CT	Several	Faster R-CNN	Organ localization
[120]	20	US	Several	RFCN	Region detection in Ultrasounds
[121]	25	E	Several	Fast R-CNN	Wireless capsule endoscopy abnormal pattern detection
[122]	12	CT	Several	Mask R-CNN	Lesion detection
[123]	22	E	Several	YOLO	Upper gastrointestinal disease
[124]	26	MRI	Several	SSD	Tuberculous and pyogenic spondylitis diagnosis

a Overview of publications that apply object detection techniques with various purposes, where PH stands for photography, US for ultrasound, A for angiography, CT for computed tomography, E for endoscopy and MRI for magnetic resonance imaging. In the header of the table, C stands for the number of citations.

Seven of the thirteen studies analyzed in this subsection focus on object detection without specifying a particular anatomical area. For the lesion detection task, two studies have been carried out. The first study [118] uses an algorithm based on Faster R-CNN and a dataset that was compiled with 23,735 lesions from 32,120 CT scans. The results show a sensitivity rate of 81.1 %, with five false positives per image. The second study [122] proposes a universal lesion detection in CT imaging. The data set was created by merging images from data sets related to the liver, lymph nodes, and the entire body, resulting in a total of over 30,000 lesions. The average sensitivity of the proposed model was reported to be 47.6 %.

Lesions in various gastrointestinal regions, including the esophagus, stomach, pylorus, duodenum, and cardia, can be detected in gastroscopic images using YOLOV3 [123]. The authors reported an mAP of 58.10 %. Additionally, this performance was compared against the of SSD and RetinaNet performances.

Organ localization is the focus of a study [119] where Faster R-CNN is used in CT imaging. In this work, the authors combined two datasets, one containing images of eleven body organs and the other containing images of twelve head organs, achieving an mAP of 73.01 % for the former and 84.78 % for the latter.

A study on region detection in ultrasounds [120] employs Faster R-CNN, and its performance is compared against SSD and RFCN. This study diagnoses various diseases, including gallbladder stones and polyps, hydronephrosis, kidney stones, renal cysts, hemangiomas, and fatty liver. RFCN emerged as the top performer of the three models with an mAP of 78.7 %, followed closely by SSD with 76.7 % and Faster R-CNN with 75.4 %.

Another study uses a Fast R-CNN based model on wireless capsule endoscopy abnormal pattern detection on more than 7000 annotated images. The final proposal achieved an mAP of 72.3 % [121].

In medical imaging, tuberculous and pyogenic spondylitis diagnosis is carried out using an SSD approach on MRI scans [124]. The results of this study show that the model achieved an AUC of 0.80, with a sensitivity of 85 % and a specificity of 67.9 %.

Additionally, the skin is also the subject of research in automatic detection systems. A study was conducted on melanoma detection using Mask R-CNN [113], to identify potential skin lesion areas and crop those areas for further analysis by a classifier algorithm. Another study analyzed scalp health and analyzed four distinct scalp hair symptoms [114]. The results showed that Faster R-CNN achieved an mAP of 91.75 %, while SSD achieved an mAP of 87.16 %.

The detection of diabetic foot ulcers is assessed using Faster R-CNN on a photographic imaging dataset consisting of 2000 training images, 200 validation images, and 2000 testing images [112]. In this study, the authors compare the performance of different algorithms in terms of mAP and other metrics, with Faster R-CNN achieving an mAP of 69.40 %, YOLOv3 an mAP of 65.60 %, Cascade DetNet an mAP of 63.94 %, YOLOv5 an mAP of 62.94 %, and EfficientDet an mAP of 56.94 %.

In CT imaging for patients with oral cancers, a DetectNet is utilized for cervical lymph node detection [117]. The sensitivity for metastatic lymph nodes was identified as 73 %, and for non-metastatic lymph nodes, a sensitivity of 52.5 % was observed.

For intracranial aneurysm detection on digital subtraction angiography, a custom object detection network is employed on vessel imaging [116]. The proposed architecture attained an accuracy of 93 % and an AUC of 0.942. In this study, the proposed network is also compared to YOLOv3 and RetinaNet, both with lower performance results.

Table 9 of Supplementary File 4 defines the data sets employed and the type of study applied to images of various topics.

## 4 Conclusion

### 4.1 Principal results

This study conducted a thorough bibliometric analysis of the relevant studies in the rapidly growing field of the application of object detection in medical image analysis. A PRISMA methodology was applied to identify 311 relevant publications in this field. Our research findings led us to understand that this is a recent area of study but one in which research is steadily increasing and has the potential to grow. Most of the publications were included in the research areas of Medicine, Computer Science, and Engineering and were published in top journals belonging to the first quartile. China, the United States, and Japan were the most productive countries, with the United States being the country with the highest level of international cooperation among these three countries. The majority of the authors with the highest number of citations were associated with only one published paper, and only six authors had five or more publications associated. In the authors’ keyword analysis, it was clear that concepts such as object detection, deep learning, artificial intelligence, and convolutional neural networks were prevalent, while in index keyword analysis, medical concepts were introduced with higher relevance.

Despite the emergence of deep learning object detection methods in 2014, medical imaging only adopted this approach in 2018, when the algorithms had evolved and demonstrated improved performance capabilities, which is a high demand in the medical field. Regardless of its relatively late arrival in the field, the introduction of object detectors in medical imaging has been modest, and only thirteen studies were published in 2018. Nonetheless, our research findings indicate this is a rapidly developing study area. Four years later, in 2022, the number of publications has increased by more than ten times, demonstrating the significant growth of the field. Additionally, it can be understood that while the establishment of object detectors in the field was initially cautious, it quickly expanded into various medical imaging techniques and a diversity of anatomical application domains.

Taking into account the results obtained in the quantitative approach, several insights can be obtained concerning the application of object detection algorithms in medical imaging, namely.

• This is a promising field of future research, weakly explored due to the late arrival of these techniques in the medical field. With the expansion and development of new and optimized frameworks of object detection, it is possible nowadays to obtain models with high performance, and with the demand of real-time detection needed for some specific fields of medicine, such as in endoscopies.
• The publications in this area have a bi-discipline nature, being almost equally distributed between Medicine-related and Computer Science fields. This dual can bring some advantages, such as comprehensive solutions and interdisciplinary insights if research is done in cross-domain collaboration. When this collaboration is not applied, there is frequently a lack of consensus regarding the terminology used in both approaches, particularly on how to evaluate the performance of those models in a manner that facilitates comparison across studies and enables the identification of best approaches and procedures for specific cases and data sets.
• China, the United States, and Japan stand as the most productive countries in this area, with the United States having the highest level of international cooperation among these countries.
• When comparing the authors' keywords analysis with the index keyword analysis, the former has a prevalence of keywords related to the algorithms themselves, such as deep learning, object detection, and medical field. In the latter analysis, medical concepts were found to be of higher relevance, suggesting the growing interest in applying advanced computational techniques to solve medical challenges, with a high number of those articles published on journals medicine- oriented.

Of the 311 papers that were analyzed, 80 of them underwent a rigorous qualitative analysis from three distinct perspectives: the algorithms utilized, the imaging modalities employed, and the anatomical areas of application. The analysis results revealed that most of the publications employed Faster R-CNN and YOLO (regardless of their specific variant) in their research, indicating that both one-stage and two-stage object detectors play a significant role, depending on the trade-off between performance and training speed. For instance, in applications where real-time predictions are a demand, such as endoscopic exams, the authors prefer one-stage algorithms because they can make faster inferences on new images. It is clear, however, that there is no consensus on the best algorithm to use since its selection depends on several factors, such as the quantity of data available, the high need for precision versus the increased demand for inference speed, and even the particular characteristics of the objects of interest in the problem at hand.

Additionally, the YOLO algorithm has gained relevance in recent years, with the various evolutions and developments across its multiple versions. However, there is still no consensus on the best algorithm to use, reinforcing the No Free Lunch Theorem concept, which states that all optimization algorithms perform identically on average when evaluated over the space of all possible problems. The strategy for one model surpassing another relies on the personalization and optimization of the algorithm for the particular task.

With regards to medical imaging modalities, object detection algorithms have been applied to a wide variety of imaging techniques, including CR scans, pathology images, and endoscopic imaging, which are among the most commonly used, and this diversity is also reflected in the broad scope of anatomical areas to which these automatic systems have been applied. Nevertheless, object detection is also applied to CT scans, photography, MRI, ultrasound, fundus photo, and angiography imaging.

Regarding the anatomical applications of object detection algorithms, digital pathology and microscopy imaging of tissues and blood smears are the most commonly explored. However, other areas are also frequently studied in this field, such as abdominal organs focusing on colon polyp detection, digestive system organs emphasizing tooth-related tasks, bone imaging for fracture identification, and breast CT scans for breast lesion detection.

In relation to the data sets employed in the various studies, a total of 125 data sets were used across the 80 studies under evaluation. Among these, 53 of them were classified as private, 63 as public, and nine were exclusively made available by the authors upon request.

On average, these data sets contained approximately 17,111 samples. However, when comparing the sample sizes based on data availability, private data sets had a notably higher average of 23,619 samples, whereas public data sets averaged only 9773 samples. Looking at the median values, 50 % of private data sets contained at least 921 images, whereas public data sets had a significantly lower median of only 700 images.

Of the 125 data sets, the minimum number of samples available was seven, and the data set with the highest number of images contained 1,024,039 samples.

In the studies evaluated, we also examined the proportion of samples used for each partition. As good practice, datasets should be divided into three non-overlapping partitions: train (to train the model), validation (to tune the hyperparameters of the model) and testing (to evaluate the model's performance on unseen data and assess generalization) [125]. Several studies follow this best practice, often splitting the dataset into two or three partitions or using external datasets (an approach that is also effective in preventing overfitting and ensuring that the test set remains independent). Although this important detail is considered in several studies, it is not consistently applied, and the proportions of each partition vary significantly, indicating a lack of standardization.

Additionally, further aspects and techniques should be considered, particularly associated with the number of samples available in each study. In many studies, the small size of the dataset highlights the need to apply sampling techniques, such as k-fold cross-validation, to maximize the use of the available data and ensure more reliable evaluations [125].

When considering whether the data sets were external or internal, out of those 125 data sets, 71 of them were categorized as external, while the remaining data were classified as internal.

Regarding the type of study applied, the majority of the studies adopted a retrospective approach, encompassing 70 out of the 80 studies. Only three studies were prospective, and two exhibited a mixed nature. In five of the 80 studies, it was not clear whether the study was retrospective or not.

Some of the public data sets were used more than once in distinct studies, namely BCCD (n = 2), CBIS-DDSM (n = 2), DDSM (n = 3), DeepLesion (n = 2), Etis-Larib (n = 2) and INBreast (n = 5).

Based on these findings, it becomes evident that the research field could significantly benefit from increased public availability of data or making data accessible upon request. Such an initiative would consequently lead to improved performance in the models utilized within these studies. Furthermore, an evident gap exists in the lack of prospective studies in the conducted research. Encouraging prospective studies is crucial for comprehending the long-term effectiveness of the developed approaches and for collecting data with the specific purpose of implementing these techniques. This approach allows researchers to exercise control over the data collection methods, ensuring data quality, reliability, and minimizing bias. Moreover, the collected data can be customized to address specific research questions in development. Such an emphasis on customization can foster further improvements and innovations in object detection techniques applied to medical imaging.

It is also important to note that some studies highlight the high capability of these automatic systems in detecting the objects of interest, sometimes with equal or higher performance than human specialists.

• The choice of an object detection algorithm is, in most cases, independent of the task itself and the screening method. As an example, Faster R-CNN is applied to the detection of gastric lesions [49], abnormality detection on the liver [48], detection of distal radius fractures [58], stroke lesion detection [69], pulmonary tuberculosis detection [82], among others. Exceptions are made when those tasks demand predictions at a faster rate or in a real-time manner, such as in endoscopies, where one-stage algorithms are preferred, such as YOLO [41,43], SSD [42,44] and RefineNet [45], in colon polyp detection.
• There is no consensus on the best algorithm to use, as it depends on various factors, including available data, precision needs, and inference speed requirements.
• Due to the bi-discipline nature of the publications mentioned above, the lack of agreement on the metrics used leads to a difficult task, if not impossible, to compare different approaches even when the same data set is used. This gap reveals the need in the research field to define ways of evaluating object detection results in an agreed manner, independently of the academic nature of the authors. Taking as an example the chest organ studies approached in this work, where pulmonary conditions such as COVID and tuberculosis are detected, some authors use precision and recall to evaluate their results on the object detection framework [81], while others focused on accuracy [79], and others on mAP [80,82], only in four distinct articles. In other tasks, other metrics are used, such as specificity [48,92,99,102,107,114,125], AUC [22,75,92,119,125],F-measure [84,89], among others.
• YOLO framework has gained importance during time, with prior versions of YOLO being associated with lower performance; however, latter versions achieve similar if not superior performance to two-stage algorithms, indicating that one-stage approaches tend to be preferred in the future. In 2018, only one study applied YOLO [41], and four studies implement Faster R-CNN [71,95,115,118]. In 2022, YOLO studies were in the number of 6 [43,50,61,67,104,109], while the Faster R-CNN algorithm was only applied in two studies [90,92]. This continuous advancement in algorithms reflects the evolving nature of the field and, consequently, the necessity of ongoing research and ad-hoc optimization for specific tasks.
• Independently of the screening methods, object detection procedures can show satisfactory results, if not better equal or even higher than human specialists, as defended in Refs. [86,92,102,108], showing the versatility and potential impact of these systems in several medical fields.

Considering these findings, it is reasonable to conclude that object detection in medical imaging is a rapidly developing research area, and this work can have a significant impact in helping to understand the direction of further research in this field.

### 4.2 Limitations

Based on two widely used academic databases, the quantitative and qualitative analysis performed in this study has employed the PRISMA methodology to screen for relevant articles. However, due to the bi-disciplinary nature of the field of study, combining both medicine and computer science, it is apparent that each discipline utilizes distinct concepts and terminologies to address similar issues. One of the primary challenges in this study was to evaluate the performance of various models across different tasks. There is a lack of consensus among researchers on which metrics should be used to make such comparisons, with some advocating for the use of commonly accepted metrics such as Average Precision (AP) and mean Average Precision (mAP) [126], while others prefer to utilize alternative metrics such as sensitivity, specificity, and accuracy.

The search strategy used in this study was focused on the title, abstract, and keywords of the publications. However, it became evident that many medical-oriented studies did not include AI concepts as a primary topic, while computer science-oriented works did not focus on medical concepts. To capture a more comprehensive set of relevant publications, future research should consider adjusting the search queries to account for the different terminology used in both disciplines. Furthermore, a more extensive comparison between the developed work should be addressed, including important details such as the data sets used, the metrics used to evaluate the models and their corresponding performance, and the remarks of the most relevant publications. Additionally, the search was limited to articles and focused on English-language publications, which may have resulted in a biased selection. This filtering may have missed relevant studies conducted in different formats and languages. Furthermore, the quantitative analysis can be further explored, including topics such as subgroup analysis for different countries. Considering these limitations, future research should aim to broaden the search scope, exploring a wider range of relevant studies in various formats and languages.

Concerning the models and the data exposed in the publications under analysis, we acknowledge the significance of addressing safety and privacy concerns. While assuming that the journals publishing the analyzed studies adhere to strict procedures that take into account those concerns, our study did not comprehensively explore the potential safety and privacy implications associated with each individual study. This concern is of utmost importance and should be highlighted, taking into account the possible implications that these models can lead when applied in real-world situations. The guidelines and future work that this particular study can induce should always be accompanied by a thorough understanding of the safety and privacy characteristics of the baseline publications. The tools and techniques developed and investigated in these studies should be viewed as supplementary procedures able to reinforce the medical expertise rather than substitutes for it.

## CRediT authorship contribution statement

**Carina Albuquerque:** Writing – review & editing, Writing – original draft, Visualization, Validation, Supervision, Software, Resources, Project administration, Methodology, Investigation, Formal analysis, Data curation, Conceptualization. **Roberto Henriques:** Writing – review & editing, Validation, Supervision, Conceptualization. **Mauro Castelli:** Writing – review & editing, Validation, Supervision, Conceptualization.

## Data availability

Data sharing is not applicable to this article as no data sets were generated or analyzed during this study.

## Declaration of generative AI in scientific writing

The authors declare no usage of generative AI in scientific writing.

## Declaration of competing interest

The authors declare that they have no known competing financial interests or personal relationships that could have appeared to influence the work reported in this paper.
