# Supplementary material for: Deep learning-based object detection algorithms in medical imaging: Systematic review
Source: Heliyon. 2024 Dec 11;11(1):e41137. doi: 10.1016/j.heliyon.2024.e41137 (PMC11699422; doi:10.1016/j.heliyon.2024.e41137)
Supplement: Multimedia component 1 [file mmc1.docx]

**Supplementary File 1**

Table 1. Article selection on Scopus and WoS, based on strings and queries.^a^

| **Database** | **N** | **Keywords** | **Strings** | **Field** | **Results** |
| --- | --- | --- | --- | --- | --- |
| WOS | #1 | Deep learning and synonyms | ("deep learning") OR ("convolutional neural network*") OR ("convolution") OR ("deep neural network*") | Topic (TS) | 299,122 |
|  | #2 | Object Detection and synonyms | ("object detection") OR ("object recognition") OR ("lesion detection") | Topic (TS) | 65,125 |
|  | #3 | Medical Imaging or synonyms | ("medical imaging") OR ("diagnostic imaging") OR ("diagnosis") OR ("computer aided diagnosis") OR ("disease*) OR ("medical computing") OR ("medical") | Topic (TS) | 7,608,363 |
|  | #4 | Segmentation | ("segment*") | Key (AK) OR Title (TI) | 228,918 |
|  | #1 AND #2 AND #3 | | | | 1,321 |
|  | (#1 AND #2 AND #3) AND NOT #4 | | | | 1,077 |
|  | (#1 AND #2 AND #3) AND NOT #4, English, Document type as "Article" | | | | 673 |
| SCOPUS | #1 | Deep learning and synonyms | ("deep learning") OR ("convolutional neural network*") OR ("convolution") OR ("deep neural network*") | TITLE-ABS-KEY | 448,227 |
|  | #2 | Object Detection and synonyms | ("object detection") OR ("object recognition") OR ("lesion detection") | TITLE-ABS-KEY | 116,390 |
|  | #3 | Medical Imaging or synonyms | ("medical imaging") OR ("diagnostic imaging") OR ("diagnosis") OR ("computer aided diagnosis") OR ("disease*) OR ("medical computing") OR ("medical") | TITLE-ABS-KEY | 15,611,842 |
|  | #4 | Segmentation | ("segment*") | Key OR Title | 479,319 |
|  | #1 AND #2 AND #3 | | | | 2,502 |
|  | (#1 AND #2 AND #3) AND NOT #4 | | | | 1,863 |
|  | (#1 AND #2 AND #3) AND NOT #4, English, Document type as "Article" | | | | 760 |

^a^ The search was conducted using both WoS and Scopus databases. Records referring to the topics of deep learning, object detection, and medical imaging in the title, abstract, or keywords were combined. Publications in which segmentation was on the topic, were excluded. The criterion for inclusion was limited to articles written in the English language.
