# Supplementary material for: Deep learning-based object detection algorithms in medical imaging: Systematic review
Source: Heliyon. 2024 Dec 11;11(1):e41137. doi: 10.1016/j.heliyon.2024.e41137 (PMC11699422; doi:10.1016/j.heliyon.2024.e41137)
Supplement: Multimedia component 2 [file mmc2.docx]

**Supplementary File 2**

Table 1. Journals with five or more publications in the area.^a^

| **Rank** | **Journal Title** | **Country** | **SJR 2021** | **No. of Citations** | **No. of Articles** | **AC** | **H Index** | **IF Scopus** | **IF WoS** |
| --- | --- | --- | --- | --- | --- | --- | --- | --- | --- |
| 1 | IEEE Access | United States | 0.93 | 111 | 17 | 6.53 | 158 | 4.342 | 3.367 |
| 2 | Scientific Reports | United Kingdom | 1.01 | 683 | 13 | 52.54 | 242 | 4.543 | 4.379 |
| 3 | PLoS ONE | United States | 0.85 | 240 | 10 | 24.00 | 367 | 3.582 | 3.24 |
| 4 | Sensors | Switzerland | 0.80 | 29 | 9 | 3.22 | 196 | 4.352 | 3.576 |
| 5 | Computers in Biology and Medicine | United Kingdom | 1.31 | 1431 | 9 | 159.00 | 102 | 7.469 | 4.589 |
| 6 | Diagnostics | Switzerland | 0.66 | 28 | 7 | 4.00 | 35 | 3.912 | 3.706 |
| **7** | IEEE Transactions of Medical Imaging | United States | 4.05 | 89 | 7 | 12.71 | 233 | 12.018 | 10.048 |
| 8 | Computer Methods and Programs in Biomedicine | Ireland | 1.33 | 107 | 6 | 17.83 | 115 | 7.639 | 5.428 |
| 9 | Biomedical Signal Processing and Control | Netherlands | 1.21 | 53 | 5 | 10.60 | 84 | 5.861 | 3.88 |
| 10 | Applied Sciences (Switzerland) | Switzerland | 0.51 | 72 | 5 | 14.40 | 75 | 3.143 | NA |
| 11 | Multimedia Tools and Applications | Netherlands | 0.72 | 19 | 5 | 3.80 | 80 | 3.158 | 2.757 |
| 12 | Medical Image Analysis | Netherlands | 4.17 | 12 | 5 | 2.40 | 143 | 15.243 | 8.545 |
| 13 | Medical Physics | United States | 1.17 | 16 | 5 | 3.20 | 189 | 4.448 | 4.071 |

^a^The SJR, or Scimago Journal Rank, is a metric that ranks journals based on the number of citations received by their articles over three years. The AC stands for the average number of citations, a ratio between the number of citations and the number of publications. The H-Index measures the number of publications and how often they are cited, reflecting the quantity and quality of a journal's publication. IF Scopus and IF WoS reflect the journal Impact Factor in each database. The results are ranked based on the number of publications.
