# Supplementary material for: Deep learning-based object detection algorithms in medical imaging: Systematic review
Source: Heliyon. 2024 Dec 11;11(1):e41137. doi: 10.1016/j.heliyon.2024.e41137 (PMC11699422; doi:10.1016/j.heliyon.2024.e41137)
Supplement: Multimedia component 3 [file mmc3.docx]

**Supplementary File 3**

Table 1. Publications with an annual citation rate higher than 10.^a^

| **Rank** | **Title (authors)** | **Annual Citation Rates** |
| --- | --- | --- |
| 1 | Automated detection of COVID-19 cases using deep neural networks with X-ray images [21] | 435.33 |
| 2 | Detecting and classifying lesions in mammograms with Deep Learning [22] | 72.80 |
| 3 | DeepLesion: Automated mining of large-scale lesion annotations and universal lesion detection with deep learning [23] | 45.60 |
| 4 | White blood cells detection and classification based on regional convolutional neural networks [24] | 31.00 |
| 5 | Artificial intelligence using convolutional neural networks for real-time detection of early esophageal neoplasia in Barrett's esophagus (with video) [25] | 30.67 |
| 6 | Evaluation of deep learning detection and classification towards computer-aided diagnosis of breast lesions in digital X-ray mammograms [26] | 30.00 |
| 7 | A deep learning approach to automatic teeth detection and numbering based on object detection in dental periapical films [27] | 29.25 |
| 8 | An improved deep learning approach for detection of thyroid papillary cancer in ultrasound images [28] | 19.60 |
| 9 | Polyp detection during colonoscopy using a regression-based convolutional neural network with a tracker [29] | 19.60 |
| 10 | An experimental study on breast lesion detection and classification from ultrasound images using deep learning architectures [30] | 19.50 |
| 11 | Evaluation of an artificial intelligence system for detecting vertical root fracture on panoramic radiography [31] | 19.33 |
| 12 | Deep learning in diabetic foot ulcers detection: A comprehensive evaluation [32] | 19.00 |
| 13 | Melanoma diagnosis using deep learning techniques on dermatoscopic images [33] | 18.50 |
| 14 | Artificial intelligence detection of distal radius fractures: a comparison between the convolutional neural network and professional assessments [34] | 18.25 |
| 15 | Automated endoscopic detection and classification of colorectal polyps using convolutional neural networks [35] | 17.33 |
| 16 | Automatic detection and classification of radiolucent lesions in the mandible on panoramic radiographs using a deep learning object detection technique [36] | 16.50 |
| 17 | An efficient real-time colonic polyp detection with YOLO algorithms trained by using negative samples and large datasets [37] | 16.00 |
| 18 | Malaria parasite detection in thick blood smear microscopic images using modified YOLOV3 and YOLOV4 models [38] | 15.50 |
| 19 | Detection of cardiac structural abnormalities in fetal ultrasound videos using deep learning [39] | 15.00 |
| 20 | Deep learning approach to peripheral leukocyte recognition [40] | 14.60 |
| 21 | Addressing class imbalance in deep learning for small lesion detection on medical images [41] | 14.33 |
| 22 | Efficient Multiple Organ Localization in CT Image Using 3D Region Proposal Network [42] | 13.50 |
| 23 | Machine learning approach of automatic identification and counting of blood cells [43] | 13.25 |
| 24 | Imaging based cervical cancer diagnostics using small object detection - generative adversarial networks [44] | 13.00 |
| 25 | Use of artificial intelligence for detection of gastric lesions by magnetically controlled capsule endoscopy [45] | 12.50 |
| 26 | Detection of masses in mammograms using a one-stage object detector based on a deep convolutional neural network [46] | 12.20 |
| 27 | Tooth detection and classification on panoramic radiographs for automatic dental chart filing: improved classification by multi-sized input data [47] | 12.00 |
| 28 | Early esophageal adenocarcinoma detection using deep learning methods [48] | 11.00 |
| 29 | Development of an artificial intelligence system using deep learning to indicate anatomical landmarks during laparoscopic cholecystectomy [49] | 10.50 |
| 30 | A novel YOLOv3-arch model for identifying cholelithiasis and classifying gallstones on CT images [50] | 10.25 |

^a^The publications are arranged in descending order based on their annual citation rate. Only articles with an annual citation rate greater than 10 are shown.

## References

**Note: The reference numbering coincides with the reference numbering in the manuscript.**

21. Ozturk T, Talo M, Yildirim EA, Baloglu UB, Yildirim O, Rajendra Acharya U. Automated detection of COVID-19 cases using deep neural networks with X-ray images. *Comput Biol Med*. 2020;121. doi:10.1016/j.compbiomed.2020.103792

22. Ribli D, Horváth A, Unger Z, Pollner P, Csabai I. Detecting and classifying lesions in mammograms with Deep Learning. *Sci Rep*. 2018;8(1). doi:10.1038/s41598-018-22437-z

23. Yan K, Wang X, Lu L, Summers RM. DeepLesion: Automated mining of large-scale lesion annotations and universal lesion detection with deep learning. *Journal of Medical Imaging*. 2018;5(3). doi:10.1117/1.JMI.5.3.036501

24. Kutlu H, Avci E, Özyurt F. White blood cells detection and classification based on regional convolutional neural networks. *Med Hypotheses*. 2020;135. doi:10.1016/j.mehy.2019.109472

25. Hashimoto R, Requa J, Dao T, et al. Artificial intelligence using convolutional neural networks for real-time detection of early esophageal neoplasia in Barrett’s esophagus (with video). *Gastrointest Endosc*. 2020;91(6):1264-1271.e1. doi:10.1016/j.gie.2019.12.049

26. Al-antari MA, Han SM, Kim TS. Evaluation of deep learning detection and classification towards computer-aided diagnosis of breast lesions in digital X-ray mammograms. *Comput Methods Programs Biomed*. 2020;196. doi:10.1016/j.cmpb.2020.105584

27. Chen H, Zhang K, Lyu P, et al. A deep learning approach to automatic teeth detection and numbering based on object detection in dental periapical films. *Sci Rep*. 2019;9(1). doi:10.1038/s41598-019-40414-y

28. Li H, Weng J, Shi Y, et al. An improved deep learning approach for detection of thyroid papillary cancer in ultrasound images. *Sci Rep*. 2018;8(1). doi:10.1038/s41598-018-25005-7

29. Zhang R, Zheng Y, Poon CCY, Shen D, Lau JYW. Polyp detection during colonoscopy using a regression-based convolutional neural network with a tracker. *Pattern Recognit*. 2018;83:209-219. doi:10.1016/j.patcog.2018.05.026

30. Cao Z, Duan L, Yang G, Yue T, Chen Q. An experimental study on breast lesion detection and classification from ultrasound images using deep learning architectures. *BMC Med Imaging*. 2019;19(1). doi:10.1186/s12880-019-0349-x

31. Fukuda M, Inamoto K, Shibata N, et al. Evaluation of an artificial intelligence system for detecting vertical root fracture on panoramic radiography. *Oral Radiol*. 2020;36(4):337-343. doi:10.1007/s11282-019-00409-x

32. Yap MH, Hachiuma R, Alavi A, et al. Deep learning in diabetic foot ulcers detection: A comprehensive evaluation. *Comput Biol Med*. 2021;135. doi:10.1016/j.compbiomed.2021.104596

33. Jojoa Acosta MF, Caballero Tovar LY, Garcia-Zapirain MB, Percybrooks WS. Melanoma diagnosis using deep learning techniques on dermatoscopic images. *BMC Med Imaging*. 2021;21(1). doi:10.1186/s12880-020-00534-8

34. Gan K, Xu D, Lin Y, et al. Artificial intelligence detection of distal radius fractures: a comparison between the convolutional neural network and professional assessments. *Acta Orthop*. 2019;90(4):394-400. doi:10.1080/17453674.2019.1600125

35. Ozawa T, Ishihara S, Fujishiro M, Kumagai Y, Shichijo S, Tada T. Automated endoscopic detection and classification of colorectal polyps using convolutional neural networks. *Therap Adv Gastroenterol*. 2020;13. doi:10.1177/1756284820910659

36. Ariji Y, Yanashita Y, Kutsuna S, et al. Automatic detection and classification of radiolucent lesions in the mandible on panoramic radiographs using a deep learning object detection technique. *Oral Surg Oral Med Oral Pathol Oral Radiol*. 2019;128(4):424-430. doi:10.1016/j.oooo.2019.05.014

37. Pacal I, Karaman A, Karaboga D, et al. An efficient real-time colonic polyp detection with YOLO algorithms trained by using negative samples and large datasets. *Comput Biol Med*. 2022;141. doi:10.1016/j.compbiomed.2021.105031

38. Abdurahman F, Fante KA, Aliy M. Malaria parasite detection in thick blood smear microscopic images using modified YOLOV3 and YOLOV4 models. *BMC Bioinformatics*. 2021;22(1). doi:10.1186/s12859-021-04036-4

39. Komatsu M, Sakai A, Komatsu R, et al. Detection of cardiac structural abnormalities in fetal ultrasound videos using deep learning. *Applied Sciences (Switzerland)*. 2021;11(1):1-12. doi:10.3390/app11010371

40. Wang Q, Bi S, Sun M, Wang Y, Wang D, Yang S. Deep learning approach to peripheral leukocyte recognition. *PLoS One*. 2018;14(6). doi:10.1371/journal.pone.0218808

41. Bria A, Marrocco C, Tortorella F. Addressing class imbalance in deep learning for small lesion detection on medical images. *Comput Biol Med*. 2020;120. doi:10.1016/j.compbiomed.2020.103735

42. Xu X, Zhou F, Liu B, Fu D, Bai X. Efficient Multiple Organ Localization in CT Image Using 3D Region Proposal Network. *IEEE Trans Med Imaging*. 2019;38(8):1885-1898. doi:10.1109/TMI.2019.2894854

43. Alam MM, Islam MT. Machine learning approach of automatic identification and counting of blood cells. *Healthc Technol Lett*. 2019;6(4):103-108. doi:10.1049/htl.2018.5098

44. Elakkiya R, Teja KSS, Jegatha Deborah L, Bisogni C, Medaglia C. Imaging based cervical cancer diagnostics using small object detection - generative adversarial networks. *Multimed Tools Appl*. 2022;81(1):191-207. doi:10.1007/s11042-021-10627-3

45. Xia J, Xia T, Pan J, et al. Use of artificial intelligence for detection of gastric lesions by magnetically controlled capsule endoscopy. *Gastrointest Endosc*. 2021;93(1):133-139.e4. doi:10.1016/j.gie.2020.05.027

46. Jung H, Kim B, Lee I, et al. Detection of masses in mammograms using a one-stage object detector based on a deep convolutional neural network. *PLoS One*. 2018;13(9). doi:10.1371/journal.pone.0203355

47. Muramatsu C, Morishita T, Takahashi R, et al. Tooth detection and classification on panoramic radiographs for automatic dental chart filing: improved classification by multi-sized input data. *Oral Radiol*. 2021;37(1):13-19. doi:10.1007/s11282-019-00418-w

48. Ghatwary N, Zolgharni M, Ye X. Early esophageal adenocarcinoma detection using deep learning methods. *Int J Comput Assist Radiol Surg*. 2019;14(4):611-621. doi:10.1007/s11548-019-01914-4

49. Tokuyasu T, Iwashita Y, Matsunobu Y, et al. Development of an artificial intelligence system using deep learning to indicate anatomical landmarks during laparoscopic cholecystectomy. *Surg Endosc*. 2021;35(4):1651-1658. doi:10.1007/s00464-020-07548-x

50. Pang S, Ding T, Qiao S, et al. A novel YOLOv3-arch model for identifying cholelithiasis and classifying gallstones on CT images. *PLoS One*. 2019;14(6). doi:10.1371/journal.pone.0217647
