# Supplementary material for: Deep learning-based object detection algorithms in medical imaging: Systematic review
Source: Heliyon. 2024 Dec 11;11(1):e41137. doi: 10.1016/j.heliyon.2024.e41137 (PMC11699422; doi:10.1016/j.heliyon.2024.e41137)
Supplement: Multimedia component 4 [file mmc4.docx]

**Supplementary File 4**

Table 1. Type of study and data sets applied in detection algorithm research on abdominal organ images.^a^

| **Reference** | **Data set** | **Privacy** | **Sample Size** | **Train** | **Valid** | **Test** | **External**  **data used** | **Study type** |
| --- | --- | --- | --- | --- | --- | --- | --- | --- |
| [29] | MICCAI 2015 | Public | 36,476 | 39.8% | 12% | 48.2% | Yes | R |
| [35] | ND | Private | 27,508 | 74.3% | 25.7% | NA | No | R |
| [37] | Etis-Larib | Public | 196 | NA | NA | 100% | Yes | R |
|  | PICCOLO WideField Dataset | Public | 3,433 | 64,2% | 26,1% | 9.7% | Yes | R |
|  | SUN Colonoscopy Video Database | Public | 158,690 | 94,7% | NA | 5,3% | Yes | R |
| [71] | WCE PillCamCOLON | Private | 301 | 70% | 10% | 20% | Yes | R |
|  | Etis-Larib | Public | 196 | NA | NA | 100% | Yes | R |
|  | CVC-ClinicDB | Public | 612 | 70% | 10% | 20% | Yes | R |
| [72] | MICCAI 2017 | Public | 37,899 | 75.9% | 11.2% | 12.9% | Yes | R |
|  | CVC colon DB | Public |  |  |  |  | Yes | R |
|  | GLRC dataset | Public |  |  |  |  | Yes | R |
|  | KUMC dataset | Public |  |  |  |  | No | R |
| [49] | ND | Private | 2,339 | NC | NC | NC | No | R |
| [50] | ND | Public | 5,986 | 66.8% | 16.5% | 16.7% | No | R |
| [73] | CBIS-DDSM | Public | 3,103 | 85% | NA | 15% | Yes | R |
| [45] | ND | Private | 1,024,039 | 80.3% | NA | 19.7% | No | R |
| [74] | ND | Private | 54,198 | 56.9% | | 43.1% | Both | Both |
| [44] | Pap-Smear Images | Public | 858 | 40% | 60% | NA | Yes | R |
|  | Colposcopy Images | Public | 7 | NC | NC | NC | Yes | R |
| [75] | MobileODT Dataset | Private | 7,094 | 44% | NA | 56% | Yes | R |
| [76] | ND | Private | 6,536 | 78% | NA | 22% | No | R |
| [39] | ND | Private | 8,182 | 74.8% | 12.3% | 12.8% | No | R |
| [77] | ND | Private | 7,032 | 88.9% | 11.1% | 20% | No | R |

^a^Overview of the data sets employed and the characteristics of the studies, including the name of the dataset, its privacy status (private or public), the sample size of the data set used, and the distribution of the samples across the training, validation, and test sets, if external data is used and the retrospective (R) or prospective (P) nature of the study. “ND” stands for non-defined, “NC” for non-clear and “NA” for non-applicable.

Table 2. Type of study and data sets applied in detection algorithm research on bone images.^a^

| **Reference** | **Data set** | **Privacy** | **Sample Size** | **Train** | **Valid** | **Test** | **External**  **data used** | **Study type** |
| --- | --- | --- | --- | --- | --- | --- | --- | --- |
| [34] | ND | Private | 2,340 | 87.2% | NA | 12.8% | No | R |
| [36] | ND | Private | 285 | 73.7% | NA | 26.3% | No | R |
| [80] | ND | Private | 1,168 | 75.4% | NA | 24.6% | No | R |
| [81] | Body Images | Private | 1,162 | 83.3% | 8.3% | 8.3% | No | R |
|  | Phantom Images | Private | 300 | 83.3% | 8,3% | 8.3% | No | P |
| [82] | ND | Private | 542 | 80% | 10% | 10% | No | R |
| [83] | ND | Private | 10,000 | 85% | 10% | 5% | No | R |
| [84] | ND | Private - U.R. | 714 | 77% | 30% | 3% | No | R |
| [85] | Digital Hand Atlas | Public | 1,369 | 90% | NA | 10% | Yes | R |
|  | ND | Private | 23,842 | 90% | NA | 10% | No | R |
| [86] | ND | Private | 941 | 60% | 20% | 20% | No | R |

^a^Overview of the data sets employed and the characteristics of the studies, including the name of the dataset, its privacy status (private, public, or U.R. – available upon request), the sample size of the data set used, and the distribution of the samples across the training, validation, and test sets, if external data is used and the retrospective (R) or prospective (P) nature of the study. “ND” stands for non-defined, and “NA” for non-applicable.

Table 3. Type of study and data sets applied in detection algorithm research on brain images.^a^

| **Reference** | **Data set** | **Privacy** | **Sample Size** | **Train** | **Valid** | **Test** | **External**  **data used** | **Study type** |
| --- | --- | --- | --- | --- | --- | --- | --- | --- |
| [88] | Brats 21 | Public | 482 | 74.7% | NA | 25.3% | Yes | R |
| [89] | ND | Private | 3,064 | 80% | NA | 20% | Yes | R |
| [90] | ND | Private | 5,668 | 80% | NA | 20% | No | R |

^a^Overview of the data sets employed and the characteristics of the studies, including the name of the dataset, its privacy status (private or public, the sample size of the data set used, and the distribution of the samples across the training, validation, and test sets, if external data is used and the retrospective (R) or prospective (P) nature of the study. “ND” stands for non-defined, and “NA” for non-applicable.

Table 4. Type of study and data sets applied in detection algorithm research on breast images.^a^

| **Reference** | **Data set** | **Privacy** | **Sample Size** | **Train** | **Valid** | **Test** | **External**  **data used** | **Study type** |
| --- | --- | --- | --- | --- | --- | --- | --- | --- |
| [22] | DDSM | Public | 2,620 | 100% | NA | NA | Yes | R |
|  | Semmelweis  University | Private - U.R. | 847 | 100% | NA | NA | No | R |
|  | INBreast | Public | 410 | NA | NA | 100% | Yes | R |
| [26] | DDSM | Public | 600 random | 70% | 20% | 10% | Yes | R |
|  | INBreast | Public | 112 random | 69.6% | 19.6% | 10.7% | Yes | R |
| [30] | ND | Private - U.R. | 1,043 | 49.4% | 33.1% | 17.5% | Yes | NC |
| [41] | INBreast | Public | 410 | 50% | NA | 50% | Yes | R |
|  | e-ophtha | Public | 381 | 50% | NA | 50% | Yes | R |
| [46] | INBreast | Public | 410 | 80% | NA | 20% | Yes | R |
|  | GURO | Private | 222 | 80% | NA | 20% | No | R |
| [92] | ND | Private | 371 | 57.7% | 19.4% | 22.9% | No | R |
| [93] | DDSM | Public | 2,620 | 72.4% | 8% | 19.5% | Yes | R |
|  | ND | Private | 40,000 | 80% | 10% | 10% | No | R |
| [94] | CBIS-DDSM | Public | 2,907 | 70% | 10% | 20% | Yes | R |
|  | INBreast | Public | 410 | 70% | 10% | 20% | Yes | R |
|  | ND | Private | 489 | 70% | 10% | 20% | No | R |

^a^Overview of the data sets employed and the characteristics of the studies, including the name of the dataset, its privacy status (private, or public or available upon request (U.R.)), the sample size of the data set used, and the distribution of the samples across the training, validation, and test sets, if external data is used and the retrospective (R) or prospective (P) nature of the study. “ND” stands for non-defined, “NC” for non-clear and “NA” for non-applicable.

Table 5. Type of study and data sets applied in detection algorithm research on chest images.^a^

| **Reference** | **Data set** | **Privacy** | **Sample Size** | **Train** | **Valid** | **Test** | **External**  **data used** | **Study type** |
| --- | --- | --- | --- | --- | --- | --- | --- | --- |
| [21] | COVID-19 | Public | 127 | 80% | 20% | NA | Yes | R |
|  | Chest-Xray8 | Public | 1,000 | 80% | 20% | NA | Yes | R |
| [95] | ND | Private | 892 | 80% | 20% | NA | No | R |
|  | Yanling Hospital | Private | 99 | NA | NA | 100% | No | R |
|  | Haikou Hospital | Private | 86 | NA | NA | 100% | No | R |
|  | NIH TB Portal | Public | 171 | NA | NA | 100% | Yes | R |
| [96] | COVIDx Dataset | Public | NC | NC | NC | NC | Yes | R |
|  | Augmented COVID-19 X-Ray Images | Public | NC | NC | NC | NC | Yes | R |
| [97] | JSRT | Private | 247 | 80% | NA | 20% | Yes | R |
|  | Montgomery Dataset | Private | 138 | 80% | NA | 20% | Yes | R |
|  | Shenzhen Dataset | Private | 662 | 80% | NA | 20% | Yes | R |
|  | FAHXJU Dataset | Private | 5,344 | 80% | NA | 20% | No | R |

^a^Overview of the data sets employed and the characteristics of the studies, including the name of the dataset, its privacy status (private, or public), the sample size of the data set used, and the distribution of the samples across the training, validation, and test sets, if external data is used and the retrospective (R) or prospective (P) nature of the study. “ND” stands for non-defined, “NC” for non-clear and “NA” for non-applicable.

Table 6. Type of study and data sets applied in detection algorithm research on images of digestive system organs.^a^

| **Reference** | **Data set** | **Privacy** | **Sample Size** | **Train** | **Valid** | **Test** | **External**  **data used** | **Study type** |
| --- | --- | --- | --- | --- | --- | --- | --- | --- |
| [25] | ND | Private | 1,832 | 75% | NA | 25% | No | R |
| [48] | Endoscopic Vision Challenge MICCAI 2015 | Public | 100 | 60% | NA | 40% | Yes | R |
| [98] | ND | Private | 641 | 80% | NA | 20% | Yes | R |
| [27] | ND | Private – U.R. | 1,250 | 64% | 16% | 20% | No | R |
| [31] | ND | Private | 1,914 | 80% | NA | 20% | No | R |
| [47] | ND | Private | 100 | 75% | NA | 25% | No | R |
| [99] | ND | Private | 100 | 70% | 10% | 20% | No | R |
| [100] | ND | Private | 591 | 90% | 10% | NA | Yes | R |
| [101] | ND | Private | 2,900 | 80% | 20% | NA | No | R |
| [102] | ND | Private – U.R. | 1,670 | 82% | 9% | 9% | No | R |

^a^Overview of the data sets employed and the characteristics of the studies, including the name of the dataset, its privacy status (private, public or available upon request (U.R.)), the sample size of the data set used, and the distribution of the samples across the training, validation, and test sets, if external data is used and the retrospective (R) or prospective (P) nature of the study. “ND” stands for non-defined, and “NA” for non-applicable.Digital pathology and microscopy.

Table 7. Type of study and data sets applied in detection algorithm research on digital pathology and microscopy images.^a^

| **Reference** | **Data set** | **Privacy** | **Sample Size** | **Train** | **Valid** | **Test** | **External**  **data used** | **Study type** |
| --- | --- | --- | --- | --- | --- | --- | --- | --- |
| [24] | BCCD | Public | 410 | 80% | NA | 20% | Yes | R |
|  | LISC | Public | 242 | 80% | NA | 20% | Yes | R |
| [104] | ND | Private | 800 | 80% | NA | 20% | No | R |
| [105] | ND | Public | 1,750 | 90% | NA | 10% | No | R |
| [106] | Dataset A | Public | 289 | 60% | 20% | 20% | No | P |
|  | Dataset B | Public | 240 | 60% | 20% | 20% | No | P |
| [107] | ND | Private | 66,627 | 90% | | 10% | No | NC |
| [38] | Malaria Dataset | Public | 1,182 | 81.7% | 9.2% | 9.1% | Yes | R |
| [40] | ND | Public | 14,700 | 92.4% | NA | 7.6% | No | R |
| [43] | BCCD | Public | 360 selected | 66.7% | 16.7% | 16.7% | Yes | R |
| [108] | ALL-IDB1 | Public | 108 | 70.4% | 19.4% | 10.2% | Yes | R |
|  | C_NMC_2019 | Public | 10,661 | 90.6% | NC | 9.4% | Yes | R |
| [109] | ND | Public | 239 | 66.7% | NA | 33.3% | No | P |
| [110] | ND | Public | 2,522 | 80% | NA | 20% | No | R |
| [111] | Malaria Dataset (BBBC041) | Public | 1364 | NC | NC | NC | Yes | R |
|  | Nuclei Dataset - 2018 Data Science Bowl (BBBC022) | Public | 700 | 85.7% | NA | 14.3% | Yes | R |
| [112] | ND | Private | 400 | 75% | NA | 25% | No | NC |
| [113] | SGZ_800× | Private | 631 | 75% | NA | 25% | No | R |
|  | SGZ_1500× | Private | 904 | 75% | NA | 25% | No | R |
|  | SGZ_1500× | Private | 938 | 75% | NA | 25% | No | R |
| [114] | ND | Private – U.R. | 17 | NC | NC | NC | No | NC |
| [115] | SVIA Dataset | Public | 130,042 | 60% | 20% | 20% | Yes | R |

^a^Overview of the data sets employed and the characteristics of the studies, including the name of the dataset, its privacy status (private, public, or available upon request (U.R.)), the sample size of the data set used, and the distribution of the samples across the training, validation, and test sets, if external data is used and the retrospective (R) or prospective (P) nature of the study. “ND” stands for non-defined, “NC” for non-clear and “NA” for non-applicable.

Table 8. Type of study and data sets applied in detection algorithm research on eye images.^a^

| **Reference** | **Data set** | **Privacy** | **Sample Size** | **Train** | **Valid** | **Test** | **External**  **data used** | **Study type** |
| --- | --- | --- | --- | --- | --- | --- | --- | --- |
| [116] | Diaretdb1 | Public | 89 | NC | NC | NC | Yes | R |
|  | MESSIDOR | Public | 1,200 | NC | NC | NC | Yes | R |
|  | HRF | Public | 45 | NC | NC | NC | Yes | R |
|  | DRHAGIS | Public | 40 | NC | NC | NC | Yes | R |
|  | ORIGA | Public | 650 | NC | NC | NC | Yes | R |
| [117] | Regular Fundus Dataset | Private | 669 | 80% | NA | 20% | Yes | R |
|  | UWF Dataset | Private | 178 | 80% | NA | 20% | Yes | R |
|  | Diabetic Retinopathy Detection | Public | 35,126 | 80% | NA | 20% | Yes | R |
|  | The 2nd diabetic retinopathy – grading and image quality estimation challenge | Public | 2,256 | 80% | NA | 20% | Yes | R |

^a^Overview of the data sets employed and the characteristics of the studies, including the name of the dataset, its privacy status (private, or public), the sample size of the data set used, and the distribution of the samples across the training, validation, and test sets, if external data is used and the retrospective (R) or prospective (P) nature of the study. “NC” stands for non-clear, and “NA” for non-applicable.

Table 9. Type of study and data sets applied in detection algorithm research across various topics.^a^

| **Reference** | **Data set** | **Privacy** | **Sample Size** | **Train** | **Valid** | **Test** | **External**  **data used** | **Study type** |
| --- | --- | --- | --- | --- | --- | --- | --- | --- |
| [32] | Diabetic Foot Ulcers Grand Challenge (DFUC2020) | Public | 4,200 | 47.6% | 4.8% | 47.6% | Yes | R |
| [33] | ISIC 2017 Challenge | Public | 2,742 | 72.8% | 5.4% | 21.8% | Yes | R |
| [118] | ND | Private | 615 | 90% | NA | 10% | Yes | NC |
| [28] | ND | Private | 4,670 | 90% | NA | 10% | No | R |
| [119] | RLS Dataset | Private – U.R. | 710 | 85.9% | NA | 14.1% | No | P |
|  | ADS Dataset | Private – U.R. | 3,786 | NC | NC | NC | No | P |
| [120] | ND | Private | 365 | 71.2% | 16.4% | 12.3% | No | R |
| [23] | DeepLesion | Public | 32,120 | 70% | 15% | 15% | Yes | R |
| [42] | Abdominal Clinical DB (MICCAI LiTS) | Public | 201 | 58.7% | 6.5% | 34.8% | Yes | R |
|  | Head Clinical DB | Private | 119 | 67.2% | 7.6% | 25.2% | Yes | R |
| [121] | ND | Private | 4,298 | 70% | NA | 30% | Yes | R |
| [122] | ND | Private | 7,200 | 50% | | 50% | Yes | R |
|  | Image Atlas from Given Imaging Incorporation | Public | 181 |  |  |  | Yes | R |
| [123] | DeepLesion | Public | 32,120 | NC | NC | NC | Yes | R |
|  | LUNA (Lung Nodule Analysis) | Public | 888 | 80% | 20% | NA | Yes | R |
|  | LITS(Liver Tumor Segmentation) | Public | 201 | 80% | 20% | NA | Yes | R |
|  | NIH-Lymph NOde | Public | 176 | 80% | 20% | NA | Yes | R |
| [124] | ND | Private | 122,524 | 66.6% | NA | 33.3% | Yes | R |
| [125] | ND | Private – U.R. | 294 | 75% | NA | 25% | No | R |

^a^Overview of the data sets employed and the characteristics of the studies, including the name of the dataset, its privacy status (private, public, or available upon request (U.R.)), the sample size of the data set used, and the distribution of the samples across the training, validation, and test sets, if external data is used and the retrospective (R) or prospective (P) nature of the study. “ND” stands for non-defined, “NC” for non-clear and “NA” for non-applicable.

## References

**Note: The reference numbering coincides with the reference numbering in the manuscript.**

21. Ozturk T, Talo M, Yildirim EA, Baloglu UB, Yildirim O, Rajendra Acharya U. Automated detection of COVID-19 cases using deep neural networks with X-ray images. *Comput Biol Med*. 2020;121. doi:10.1016/j.compbiomed.2020.103792

22. Ribli D, Horváth A, Unger Z, Pollner P, Csabai I. Detecting and classifying lesions in mammograms with Deep Learning. *Sci Rep*. 2018;8(1). doi:10.1038/s41598-018-22437-z

23. Yan K, Wang X, Lu L, Summers RM. DeepLesion: Automated mining of large-scale lesion annotations and universal lesion detection with deep learning. *Journal of Medical Imaging*. 2018;5(3). doi:10.1117/1.JMI.5.3.036501

24. Kutlu H, Avci E, Özyurt F. White blood cells detection and classification based on regional convolutional neural networks. *Med Hypotheses*. 2020;135. doi:10.1016/j.mehy.2019.109472

25. Hashimoto R, Requa J, Dao T, et al. Artificial intelligence using convolutional neural networks for real-time detection of early esophageal neoplasia in Barrett’s esophagus (with video). *Gastrointest Endosc*. 2020;91(6):1264-1271.e1. doi:10.1016/j.gie.2019.12.049

26. Al-antari MA, Han SM, Kim TS. Evaluation of deep learning detection and classification towards computer-aided diagnosis of breast lesions in digital X-ray mammograms. *Comput Methods Programs Biomed*. 2020;196. doi:10.1016/j.cmpb.2020.105584

27. Chen H, Zhang K, Lyu P, et al. A deep learning approach to automatic teeth detection and numbering based on object detection in dental periapical films. *Sci Rep*. 2019;9(1). doi:10.1038/s41598-019-40414-y

28. Li H, Weng J, Shi Y, et al. An improved deep learning approach for detection of thyroid papillary cancer in ultrasound images. *Sci Rep*. 2018;8(1). doi:10.1038/s41598-018-25005-7

29. Zhang R, Zheng Y, Poon CCY, Shen D, Lau JYW. Polyp detection during colonoscopy using a regression-based convolutional neural network with a tracker. *Pattern Recognit*. 2018;83:209-219. doi:10.1016/j.patcog.2018.05.026

30. Cao Z, Duan L, Yang G, Yue T, Chen Q. An experimental study on breast lesion detection and classification from ultrasound images using deep learning architectures. *BMC Med Imaging*. 2019;19(1). doi:10.1186/s12880-019-0349-x

31. Fukuda M, Inamoto K, Shibata N, et al. Evaluation of an artificial intelligence system for detecting vertical root fracture on panoramic radiography. *Oral Radiol*. 2020;36(4):337-343. doi:10.1007/s11282-019-00409-x

32. Yap MH, Hachiuma R, Alavi A, et al. Deep learning in diabetic foot ulcers detection: A comprehensive evaluation. *Comput Biol Med*. 2021;135. doi:10.1016/j.compbiomed.2021.104596

33. Jojoa Acosta MF, Caballero Tovar LY, Garcia-Zapirain MB, Percybrooks WS. Melanoma diagnosis using deep learning techniques on dermatoscopic images. *BMC Med Imaging*. 2021;21(1). doi:10.1186/s12880-020-00534-8

34. Gan K, Xu D, Lin Y, et al. Artificial intelligence detection of distal radius fractures: a comparison between the convolutional neural network and professional assessments. *Acta Orthop*. 2019;90(4):394-400. doi:10.1080/17453674.2019.1600125

35. Ozawa T, Ishihara S, Fujishiro M, Kumagai Y, Shichijo S, Tada T. Automated endoscopic detection and classification of colorectal polyps using convolutional neural networks. *Therap Adv Gastroenterol*. 2020;13. doi:10.1177/1756284820910659

36. Ariji Y, Yanashita Y, Kutsuna S, et al. Automatic detection and classification of radiolucent lesions in the mandible on panoramic radiographs using a deep learning object detection technique. *Oral Surg Oral Med Oral Pathol Oral Radiol*. 2019;128(4):424-430. doi:10.1016/j.oooo.2019.05.014

37. Pacal I, Karaman A, Karaboga D, et al. An efficient real-time colonic polyp detection with YOLO algorithms trained by using negative samples and large datasets. *Comput Biol Med*. 2022;141. doi:10.1016/j.compbiomed.2021.105031

38. Abdurahman F, Fante KA, Aliy M. Malaria parasite detection in thick blood smear microscopic images using modified YOLOV3 and YOLOV4 models. *BMC Bioinformatics*. 2021;22(1). doi:10.1186/s12859-021-04036-4

39. Komatsu M, Sakai A, Komatsu R, et al. Detection of cardiac structural abnormalities in fetal ultrasound videos using deep learning. *Applied Sciences (Switzerland)*. 2021;11(1):1-12. doi:10.3390/app11010371

40. Wang Q, Bi S, Sun M, Wang Y, Wang D, Yang S. Deep learning approach to peripheral leukocyte recognition. *PLoS One*. 2018;14(6). doi:10.1371/journal.pone.0218808

41. Bria A, Marrocco C, Tortorella F. Addressing class imbalance in deep learning for small lesion detection on medical images. *Comput Biol Med*. 2020;120. doi:10.1016/j.compbiomed.2020.103735

42. Xu X, Zhou F, Liu B, Fu D, Bai X. Efficient Multiple Organ Localization in CT Image Using 3D Region Proposal Network. *IEEE Trans Med Imaging*. 2019;38(8):1885-1898. doi:10.1109/TMI.2019.2894854

43. Alam MM, Islam MT. Machine learning approach of automatic identification and counting of blood cells. *Healthc Technol Lett*. 2019;6(4):103-108. doi:10.1049/htl.2018.5098

44. Elakkiya R, Teja KSS, Jegatha Deborah L, Bisogni C, Medaglia C. Imaging based cervical cancer diagnostics using small object detection - generative adversarial networks. *Multimed Tools Appl*. 2022;81(1):191-207. doi:10.1007/s11042-021-10627-3

45. Xia J, Xia T, Pan J, et al. Use of artificial intelligence for detection of gastric lesions by magnetically controlled capsule endoscopy. *Gastrointest Endosc*. 2021;93(1):133-139.e4. doi:10.1016/j.gie.2020.05.027

46. Jung H, Kim B, Lee I, et al. Detection of masses in mammograms using a one-stage object detector based on a deep convolutional neural network. *PLoS One*. 2018;13(9). doi:10.1371/journal.pone.0203355

47. Muramatsu C, Morishita T, Takahashi R, et al. Tooth detection and classification on panoramic radiographs for automatic dental chart filing: improved classification by multi-sized input data. *Oral Radiol*. 2021;37(1):13-19. doi:10.1007/s11282-019-00418-w

48. Ghatwary N, Zolgharni M, Ye X. Early esophageal adenocarcinoma detection using deep learning methods. *Int J Comput Assist Radiol Surg*. 2019;14(4):611-621. doi:10.1007/s11548-019-01914-4

49. Tokuyasu T, Iwashita Y, Matsunobu Y, et al. Development of an artificial intelligence system using deep learning to indicate anatomical landmarks during laparoscopic cholecystectomy. *Surg Endosc*. 2021;35(4):1651-1658. doi:10.1007/s00464-020-07548-x

50. Pang S, Ding T, Qiao S, et al. A novel YOLOv3-arch model for identifying cholelithiasis and classifying gallstones on CT images. *PLoS One*. 2019;14(6). doi:10.1371/journal.pone.0217647

71. Souaidi M, Ansari ME. A New Automated Polyp Detection Network MP-FSSD in WCE and Colonoscopy Images Based Fusion Single Shot Multibox Detector and Transfer Learning. *IEEE Access*. 2022;10:47124-47140. doi:10.1109/ACCESS.2022.3171238

72. Li K, Fathan MI, Patel K, et al. Colonoscopy polyp detection and classification: Dataset creation and comparative evaluations. *PLoS One*. 2021;16(8 August). doi:10.1371/journal.pone.0255809

73. Xi P, Guan H, Shu C, Borgeat L, Goubran R. An integrated approach for medical abnormality detection using deep patch convolutional neural networks. *Visual Computer*. 2020;36(9):1869-1882. doi:10.1007/s00371-019-01775-7

74. Wu L, Xu M, Jiang X, et al. Real-time artificial intelligence for detecting focal lesions and diagnosing neoplasms of the stomach by white-light endoscopy (with videos). *Gastrointest Endosc*. 2022;95(2):269-280.e6. doi:10.1016/j.gie.2021.09.017

75. Xue Z, Novetsky AP, Einstein MH, et al. A demonstration of automated visual evaluation of cervical images taken with a smartphone camera. *Int J Cancer*. 2020;147(9):2416-2423. doi:10.1002/ijc.33029

76. Bai B, Du Y, Liu P, Sun P, Li P, Lv Y. Detection of cervical lesion region from colposcopic images based on feature reselection. *Biomed Signal Process Control*. 2020;57. doi:10.1016/j.bspc.2019.101785

77. Dong J, Liu S, Liao Y, et al. A Generic Quality Control Framework for Fetal Ultrasound Cardiac Four-Chamber Planes. *IEEE J Biomed Health Inform*. 2020;24(4):931-942. doi:10.1109/JBHI.2019.2948316

80. Kuwana R, Ariji Y, Fukuda M, et al. Performance of deep learning object detection technology in the detection and diagnosis of maxillary sinus lesions on panoramic radiographs. *Dentomaxillofacial Radiology*. 2020;50(1). doi:10.1259/dmfr.20200171

81. Huang Q, Luo H, Yang C, et al. Anatomical prior based vertebra modelling for reappearance of human spines. *Neurocomputing*. 2022;500:750-760. doi:10.1016/j.neucom.2022.05.033

82. Hardalaç F, Uysal F, Peker O, et al. Fracture Detection in Wrist X-ray Images Using Deep Learning-Based Object Detection Models. *Sensors*. 2022;22(3). doi:10.3390/s22031285

83. Rouzrokh P, Ramazanian T, Wyles CC, et al. Deep Learning Artificial Intelligence Model for Assessment of Hip Dislocation Risk Following Primary Total Hip Arthroplasty From Postoperative Radiographs. *Journal of Arthroplasty*. 2021;36(6):2197-2203.e3. doi:10.1016/j.arth.2021.02.028

84. Tsai JY, Hung IYJ, Guo YL, et al. Lumbar Disc Herniation Automatic Detection in Magnetic Resonance Imaging Based on Deep Learning. *Front Bioeng Biotechnol*. 2021;9. doi:10.3389/fbioe.2021.708137

85. Liang B, Zhai Y, Tong C, et al. A deep automated skeletal bone age assessment model via region-based convolutional neural network. *Future Generation Computer Systems*. 2019;98:54-59. doi:10.1016/j.future.2019.01.057

86. Li YC, Chen HH, Horng-Shing Lu H, Hondar Wu HT, Chang MC, Chou PH. Can a Deep-learning Model for the Automated Detection of Vertebral Fractures Approach the Performance Level of Human Subspecialists? *Clin Orthop Relat Res*. 2021;479(7):1598-1612. doi:10.1097/CORR.0000000000001685

88. Shelatkar T, Urvashi D, Shorfuzzaman M, Alsufyani A, Lakshmanna K. Diagnosis of Brain Tumor Using Light Weight Deep Learning Model with Fine-Tuning Approach. *Comput Math Methods Med*. 2022;2022. doi:10.1155/2022/2858845

89. Montalbo FJP. A computer-aided diagnosis of brain tumors using a fine-tuned yolo-based model with transfer learning. *KSII Transactions on Internet and Information Systems*. 2020;14(12):4816-4834. doi:10.3837/tiis.2020.12.011

90. Zhang S, Xu S, Tan L, Wang H, Meng J. Stroke Lesion Detection and Analysis in MRI Images Based on Deep Learning. *J Healthc Eng*. 2021;2021. doi:10.1155/2021/5524769

92. Adachi M, Fujioka T, Mori M, et al. Detection and diagnosis of breast cancer using artificial intelligence based assessment of maximum intensity projection dynamic contrast-enhanced magnetic resonance images. *Diagnostics*. 2020;10(5). doi:10.3390/diagnostics10050330

93. Liu Y, Zhang F, Chen C, Wang S, Wang Y, Yu Y. Act Like a Radiologist: Towards Reliable Multi-View Correspondence Reasoning for Mammogram Mass Detection. *IEEE Trans Pattern Anal Mach Intell*. 2022;44(10):5947-5961. doi:10.1109/TPAMI.2021.3085783

94. Baccouche A, Garcia-Zapirain B, Olea CC, Elmaghraby AS. Breast lesions detection and classification via YOLO-based fusion models. *Computers, Materials and Continua*. 2021;69(1):1407-1425. doi:10.32604/cmc.2021.018461

95. Yan C, Wang L, Lin J, et al. A fully automatic artificial intelligence–based CT image analysis system for accurate detection, diagnosis, and quantitative severity evaluation of pulmonary tuberculosis. *Eur Radiol*. 2022;32(4):2188-2199. doi:10.1007/s00330-021-08365-z

96. Mahajan S, Raina A, Gao XZ, Pandit AK. COVID-19 detection using hybrid deep learning model in chest x-rays images. *Concurr Comput*. 2022;34(5). doi:10.1002/cpe.6747

97. Xie Y, Wu Z, Han X, et al. Computer-Aided System for the Detection of Multicategory Pulmonary Tuberculosis in Radiographs. *J Healthc Eng*. 2020;2020. doi:10.1155/2020/9205082

98. Li X, Zhang Y, Cui Q, Yi X, Zhang Y. Tooth-Marked Tongue Recognition Using Multiple Instance Learning and CNN Features. *IEEE Trans Cybern*. 2019;49(2):380-387. doi:10.1109/TCYB.2017.2772289Y

99. Thanathornwong B, Suebnukarn S. Automatic detection of periodontal compromised teeth in digital panoramic radiographs using faster regional convolutional neural networks. *Imaging Sci Dent*. 2020;50(2):169-174. doi:10.5624/isd.2020.50.2.169

100. Estai M, Tennant M, Gebauer D, et al. Deep learning for automated detection and numbering of permanent teeth on panoramic images. *Dentomaxillofacial Radiology*. 2022;51(2). doi:10.1259/dmfr.20210296

101. Chen H, Li H, Zhao Y, Zhao J, Wang Y. Dental disease detection on periapical radiographs based on deep convolutional neural networks. *Int J Comput Assist Radiol Surg*. 2021;16(4):649-661. doi:10.1007/s11548-021-02319-y

102. Liu M, Wang S, Chen H, Liu Y. A pilot study of a deep learning approach to detect marginal bone loss around implants. *BMC Oral Health*. 2022;22(1). doi:10.1186/s12903-021-02035-8

104. Kawazoe Y, Shimamoto K, Yamaguchi R, et al. Faster R-CNN-based glomerular detection in multistained human whole slide images. *J Imaging*. 2018;4(7). doi:10.3390/jimaging4070091

105. Kassis T, Hernandez-Gordillo V, Langer R, Griffith LG. OrgaQuant: Human Intestinal Organoid Localization and Quantification Using Deep Convolutional Neural Networks. *Sci Rep*. 2019;9(1):1-7. doi:10.1038/s41598-019-48874-y

106. Rosati R, Romeo L, Silvestri S, Marcheggiani F, Tiano L, Frontoni E. Faster R-CNN approach for detection and quantification of DNA damage in comet assay images. *Comput Biol Med*. 2020;123. doi:10.1016/j.compbiomed.2020.103912

107. Xiang Y, Sun W, Pan C, Yan M, Yin Z, Liang Y. A novel automation-assisted cervical cancer reading method based on convolutional neural network. *Biocybern Biomed Eng*. 2020;40(2):611-623. doi:10.1016/j.bbe.2020.01.016

108. Khandekar R, Shastry P, Jaishankar S, Faust O, Sampathila N. Automated blast cell detection for Acute Lymphoblastic Leukemia diagnosis. *Biomed Signal Process Control*. 2021;68. doi:10.1016/j.bspc.2021.102690

109. Manescu P, Shaw MJ, Elmi M, et al. Expert-level automated malaria diagnosis on routine blood films with deep neural networks. *Am J Hematol*. 2020;95(8):883-891. doi:10.1002/ajh.25827

110. Koga S, Ikeda A, Dickson DW. Deep learning-based model for diagnosing Alzheimer’s disease and tauopathies. *Neuropathol Appl Neurobiol*. 2022;48(1). doi:10.1111/nan.12759

111. Hung J, Goodman A, Ravel D, et al. Keras R-CNN: Library for cell detection in biological images using deep neural networks. *BMC Bioinformatics*. 2020;21(1). doi:10.1186/s12859-020-03635-x

112. Mochalova EN, Kotov IA, Rozenberg JM, Nikitin MP. Precise Quantitative Analysis of Cell Targeting by Particle-Based Agents Using Imaging Flow Cytometry and Convolutional Neural Network. *Cytometry Part A*. 2020;97(3):279-287. doi:10.1002/cyto.a.23939

113. Yu W, Xue Y, Knoops R, et al. Automated diatom searching in the digital scanning electron microscopy images of drowning cases using the deep neural networks. *Int J Legal Med*. 2021;135(2):497-508. doi:10.1007/s00414-020-02392-z

114. Marzahl C, Aubreville M, Bertram CA, et al. Deep Learning-Based Quantification of Pulmonary Hemosiderophages in Cytology Slides. *Sci Rep*. 2020;10(1). doi:10.1038/s41598-020-65958-2

115. Chen A, Li C, Zou S, et al. SVIA dataset: A new dataset of microscopic videos and images for computer-aided sperm analysis. *Biocybern Biomed Eng*. 2022;42(1):204-214. doi:10.1016/j.bbe.2021.12.010

116. Nazir T, Irtaza A, Javed A, Malik H, Hussain D, Naqvi RA. Retinal image analysis for diabetes-based eye disease detection using deep learning. *Applied Sciences (Switzerland)*. 2020;10(18). doi:10.3390/APP10186185

117. Ju L, Wang X, Zhao X, Bonnington P, Drummond T, Ge Z. Leveraging Regular Fundus Images for Training UWF Fundus Diagnosis Models via Adversarial Learning and Pseudo-Labeling. *IEEE Trans Med Imaging*. 2021;40(10):2911-2925. doi:10.1109/TMI.2021.3056395

118. Chang WJ, Chen LB, Chen MC, Chiu YC, Lin JY. ScalpEye: A Deep Learning-Based Scalp Hair Inspection and Diagnosis System for Scalp Health. *IEEE Access*. 2020;8:134826-134837. doi:10.1109/ACCESS.2020.3010847

119. Duan H, Huang Y, Liu L, Dai H, Chen L, Zhou L. Automatic detection on intracranial aneurysm from digital subtraction angiography with cascade convolutional neural networks. *Biomed Eng Online*. 2019;18(1). doi:10.1186/s12938-019-0726-2

120. Ariji Y, Fukuda M, Nozawa M, et al. Automatic detection of cervical lymph nodes in patients with oral squamous cell carcinoma using a deep learning technique: a preliminary study. *Oral Radiol*. 2021;37(2):290-296. doi:10.1007/s11282-020-00449-8

121. Zeng X, Wen L, Liu B, Qi X. Deep learning for ultrasound image caption generation based on object detection. *Neurocomputing*. 2020;392:132-141. doi:10.1016/j.neucom.2018.11.114

122. Lan L, Ye C, Wang C, Zhou S. Deep Convolutional Neural Networks for WCE Abnormality Detection: CNN Architecture, Region Proposal and Transfer Learning. *IEEE Access*. 2019;7:30017-30032. doi:10.1109/ACCESS.2019.2901568

123. Yan K, Cai J, Zheng Y, et al. Learning from Multiple Datasets with Heterogeneous and Partial Labels for Universal Lesion Detection in CT. *IEEE Trans Med Imaging*. 2021;40(10):2759-2770. doi:10.1109/TMI.2020.3047598

124. Ding S, Li L, Li Z, Wang H, Zhang Y. Smart electronic gastroscope system using a cloud–edge collaborative framework. *Future Generation Computer Systems*. 2019;100:395-407. doi:10.1016/j.future.2019.04.031

125. Kim K, Kim S, Lee YH, Lee SH, Lee HS, Kim S. Performance of the deep convolutional neural network based magnetic resonance image scoring algorithm for differentiating between tuberculous and pyogenic spondylitis. *Sci Rep*. 2018;8(1). doi:10.1038/s41598-018-31486-3
